# A Comprehensive Study of the WRKY Transcription Factor Family in Strawberry

**DOI:** 10.3390/plants11121585

**Published:** 2022-06-15

**Authors:** José Garrido-Gala, José-Javier Higuera, Antonio Rodríguez-Franco, Juan Muñoz-Blanco, Francisco Amil-Ruiz, José L. Caballero

**Affiliations:** 1 Phytoplant Research SLU, 14071 Córdoba, Spain; j.garrido@phytoplant.es; 2 Departamento de Bioquímica y Biología Molecular, Campus Universitario de Rabanales y Campus de Excelencia Internacional Agroalimentario ceiA3, Edificio Severo Ochoa-C6, Universidad de Córdoba, 14071 Córdoba, Spain; b92hisoj@uco.es (J.-J.H.); bb1rofra@uco.es (A.R.-F.); bb1mublj@uco.es (J.M.-B.); 3 Unidad de Bioinformática, Servicio Central de Apoyo a la Investigación (SCAI), Universidad de Córdoba, 14071 Córdoba, Spain; b72amruf@uco.es

**Keywords:** strawberry, WRKY family, homeolog, paralog, differential expression, ripening, pathogens

## Abstract

WRKY transcription factors play critical roles in plant growth and development or stress responses. Using up-to-date genomic data, a total of 64 and 257 WRKY genes have been identified in the diploid woodland strawberry, *Fragaria vesca*, and the more complex allo-octoploid commercial strawberry, *Fragaria* × *ananassa* cv. Camarosa, respectively. The completeness of the new genomes and annotations has enabled us to perform a more detailed evolutionary and functional study of the strawberry WRKY family members, particularly in the case of the cultivated hybrid, in which homoeologous and paralogous *FaWRKY* genes have been characterized. Analysis of the available expression profiles has revealed that many strawberry *WRKY* genes show preferential or tissue-specific expression. Furthermore, significant differential expression of several *FaWRKY* genes has been clearly detected in fruit receptacles and achenes during the ripening process and pathogen challenged, supporting a precise functional role of these strawberry genes in such processes. Further, an extensive analysis of predicted development, stress and hormone-responsive cis-acting elements in the strawberry WRKY family is shown. Our results provide a deeper and more comprehensive knowledge of the *WRKY* gene family in strawberry.

## 1. Introduction

The WRKY superfamily of transcription factors (WRKY TFs) [1] has an early origin in primitive eukaryotes, with later expansion and evolution in the green lineage driven by extensive tandem and segmental duplication events [2,3,4], thus becoming one of the largest TF families in higher plants [5]. The WRKY TFs are involved in the regulation of various physiological and developmental processes, such as senescence [6,7,8], stem elongation and seed development [9] and flowering [10]. They are also key players in responses to abiotic stresses [11] as well as to biotic stresses, wherein they seem to play major roles in plant immunity [5,12,13].

WRKY TFs regulate their target’s expression by binding to a specific cis-element known as a W-box, with the minimal consensus sequence TTGACY (where Y = C/T), although adjacent sequences are also involved in the binding site preferences [14]. DNA binding by WRKY proteins is mediated by a highly conserved DNA binding domain named the WRKY domain (WD). It is about 60 amino acids long and contains an N-terminal core motif, formed by the almost invariant heptapeptide WRKYGQK, and a distinctive C-terminal zinc-finger (Znf), both required for the DNA binding activity [15]. The amino acids constituting the WRKY core motif are essential for the DNA-binding activity and recognition specificity. Thus amino acid substitutions in this motif can affect the binding to the W-box [14,16,17]. Variations in this conserved sequence have been found in WRKY proteins from different plant species [3], with functional and binding activity characterization in some cases. WRKYs harboring divergent core motifs can bind to novel sequences that deviate from the consensus W-box. For example, NtWRKY12 contains a WRKYGKK core motif, interacting with the WK-box (TTTTCCAC) but not to the consensus W-box [18]. On the other hand, modified motifs can also be unable to bind to the W-box sequence, as GmWRKY167, containing the WRKYEDK core motif [19]. However, it is unclear if the binding specificity depends exclusively on the WRKY core motif. OsWRKY7, which contains the WRKYGKK sequence, binds to the W-box but not with the WK-box [20]. In addition, AtWRKY70, which harbors the conserved WRKYGQK core motif, can bind to the consensus W-box, as well as the novel WT-box (YGACTTTT) [21].

The classical WRKY classification in the three groups was based on both the number of WDs and the pattern of the Znf domain [1]. Group I WRKY proteins are the only ones that have two WDs (I-NT, I-CT), with a C2H2 Znf pattern (CX_4–5_CX_22–23_HXH) shared with group II WRKYs. Group II was further divided into subgroups IIa, IIb, IIc, IId and IIe based on the differences in their amino acid sequences from their WD. Group III WRKY proteins contain a WD with a C2HC Znf pattern (CX_7_CX_23_HXC). Later studies have proposed a new phylogenetic classification into four major groups (Group I + IIc, Group IIa + IIb, Group IId + IIe and Group III), as well as new hypotheses for the evolution of WRKY genes [22]. The WRKY proteins can also include additional domains. Notoriously, the R protein-WRKY family contains several typical domains of R-proteins, such as Toll-interleukin 1 receptor (TIR), leucine-rich repeat (LRR) and NB-ARC, which are associated with WDs from Group I, Group II and Group III members. Such chimeric genes have been found in multiple plant genomes; however, they are not widespread in plants. Instead, R protein-*WRKY* genes appear to have evolved on multiple independent occasions as a result of particular genomic rearrangements within specific plant lineages [22].

The genus *Fragaria* (*Rosaceae*) comprises about 24 species worldwide, with different geographical distributions and ploidy levels, from diploid to decaploid [23]. The modern cultivated octoploid strawberry (*Fragaria* × *ananassa* Duch.) is presumably the most economically important soft berry, with a world production exceeding 8.86 Mt and 384,668 ha harvested (FAOSTAT, 2020). In recent years, many efforts have been made to unravel the genetic background of this species to be used as a molecular breeding tool to identify traits and associated genes of interest for the genetic improvement of this valuable crop. The genome of the diploid (2n = 14) strawberry *Fragaria vesca* (Fv) was sequenced and published for the first time in 2011 [24] and proposed as a gateway to functional studies of genes within the *Rosaceae*, particularly for the cultivated strawberry. The first assembly versions were improved and reannotated [25,26,27]. Recently, it has been sequenced de novo using third-generation PacBio Single Molecule, Real-Time (SMRT) sequencing technology and an overall improvement over previous versions has been achieved [28]. Moreover, gene models and genome annotation have been recently updated [29]. *Fragaria* × *ananassa* (Fa) is an allo-octoploid hybrid (2n = 8x = 56), which originated around 300 years ago from interspecific crosses of the also octoploids *F. virginiana* and *F. chiloensis*. Recently, the genome of *Fragaria* × *ananassa* cv. Camarosa has been completely sequenced and annotated, revealing its diploid progenitor species: *F. vesca* (subsp. *bracheata*), *F. iinumae*, *F. nipponica* and *F. viridis* [30], although the contribution of the last two species has been debated [31,32,33]. Remarkably, this study has found that the subgenome contributed by Fv is dominant and has replaced large portions of the submissive ones through homoeologous exchanges.

The emergence of all these new data represents a valuable opportunity to conduct new and more comprehensive evolutionary analyses on strawberry, as well as greatly facilitate functional studies to further unravel the roles of members of the WRKY family in regulating the physiology of the strawberry, particularly in key aspects such as fruit ripening and biotic stress. This is especially relevant for the cultivated strawberry, in which many previous studies have been carried out with the limited availability of genetic data on the structure and nucleotide sequence of the octoploid genome, but taking advantage of the synteny and high sequence identity with Fv to use its reference genome as an “anchor” between both species [34,35,36,37]. For *F. vesca*, we describe extensive new findings not previously covered by Wei et al. (2016) [38] and Zhou et al. (2016) [39]. Moreover, a complete description of the *F. ananassa* WRKY members, defining the homoeologs and paralogs genes, is presented for the first time. We have further concentrated the expression analysis on fruit ripening, and biotic responses and a comprehensive study regarding the number and density of predicted development, stress and hormone responsive cis-acting elements in the WRKY TFs is shown together with their exon-intron distribution. In consequence, this study updates and expands our knowledge of the members of diploid and octoploid strawberry WRKY TF family, their evolutionary history and their potential roles in specific strawberry tissues and important biological processes such as fruit ripening and defense responses against pathogens.

## 2. Results and Discussion

### 2.1. WRKY Members of F. vesca and F. ananassa

The FvWRKY family has been described before using an earlier annotation [38,39]. Here, we have used the latest Fv genome and annotation versions available, which combine a higher quality reference genome [28] and high-fidelity gene models with RNA-seq support using expression data from different *F. vesca* accessions, tissues and fruit developmental stages [29]. A comprehensive list of the FvWRKY members across different Fv genome annotations is also provided (Table S1). A total of 64 *FvWRKY* coding genes and their splicing forms (SFs) were confirmed, then named according to their chromosomal locations (Table S2). Some relevant properties of the FvWRKY proteins and their predicted subcellular location are also listed. GRAVY (grand average of hydropathy) values are below 0, indicating that FvWRKYs are hydrophilic and more likely globular-shaped, while LOCALIZER predicted the protein location inside the nucleus for most FvWRKYs.

FvWRKY proteins were classified into groups I, II and III according to their WDs [1]. Core WRKY motif modifications were detected in FvWRKY3 and -8 (WRKYGKK), FvWRKY21 (WKKYGQK), FvWRKY35 (WTKYDQR) and FvWRKY55 (WREYDQR). A more drastic modification is found in FvWRKY37, in which the core motif is found truncated and reduced to WRK. Moreover, differences in some *FvWRKYs* splicing forms (SF) were observed, affecting the nature of the encoded proteins. Thus, one SF from *FvWRKY20*, -*26* and -*43* (Group I) encode for WRKY proteins that have lost their I-NT WDs, while some SF from several *FvWRKYs* encode proteins containing incomplete WDs. Additionally, one SF from *FvWRKY54* and -*62* have lost their WDs completely. The regulation of all these alternative transcripts and whether they are efficiently translated into functional proteins remains to be studied.

**Figure 1:**
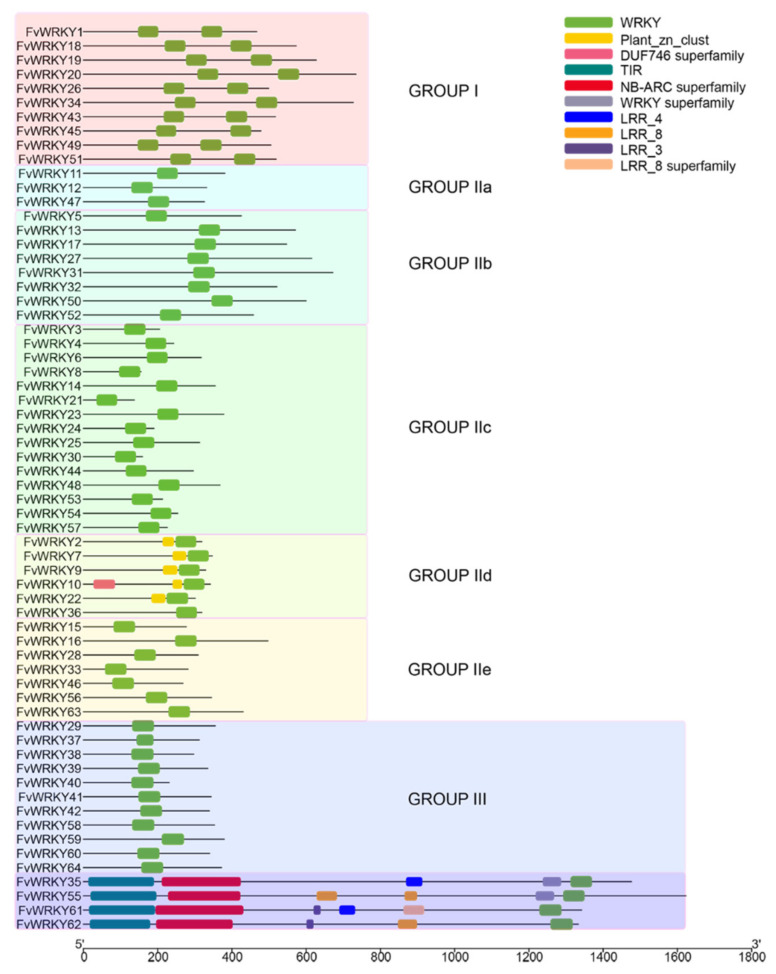
Protein domains (PFAM v32.0) found in FvWRKY proteins. Only the largest splice forms are represented. See Table S2 for more details.

Additional motifs were also found in some of the FvWRKY proteins (Figure 1). These include a plant zinc cluster domain in FvWRKY2, -7, -9, -10 and -22. Further, TIR, NB-ARC and leucine-rich repeat motifs (LRR) harbored by the FvRWRKY subfamily [22], consisting of FvWRKY35, -55, -61 and -62. Moreover, FvWRKY35 and -55 exhibit an additional WRKY-like domain, lacking the core motif but retaining the Znf portion. Additionally, the loss of the WD in the predicted *FvWRKY62.t6* splicing form would turn the translated product into a TIR-NBS-LRR protein.

The recent *Fragaria* × *ananassa* Camarosa (Fa) Genome Assembly v1.0 and annotation v1.0.a1 were used to identify the *FaWRKY* candidates distributed among the four diploid subgenomes, which compose this allo-octoploid [30]. A total of 255 FaWRKY were found by HMMER, and the presence of WD was confirmed in CDD. Homology and shared synteny with the *FvWRKY* genes were investigated using SynMap2. An additional gene (*snap_masked-Fvb3-3-processed-gene-269.16*) showed shared synteny with FvWRKY20. This Fa gene was not detected by the HMMER search due to an incomplete protein sequence in the source dataset. FGENESH (www.softberry.com, accessed on 14 April 2022) [40] was used to predict a revised protein sequence, using the source mRNA as input and specific gene-finding parameters for Fv. The corrected protein sequence showed two WDs, and this gene was named *FaWRKY20A* (Figure S1). Likewise, shared synteny was found between another gene (*maker-Fvb3-1-snap-gene-3.50*) and *FvWRKY21*. A genomic DNA track containing this gene (Fvb3-1:336748..339128) was loaded in FGENESH to predict newly revised mRNA and protein sequences and the new *FaWRKY* member was named *FaWRKY21C* (Figure S2). Therefore, a total of 257 *FaWRKY* genes were found in our analysis. Two more *FaWRKYs*, *maker-Fvb3-2-snap-gene-310.32* (*FaWRKY21B*) and *maker-Fvb6-2-snap-gene-308.69* (*FaWRKY51A.2*), were detected to have incorrect protein predicted sequences in the source dataset. Genomic DNA fragments containing each gene (Fvb3-2:31015005..31018524 and Fvb6-2:30857826..30862505, respectively) were loaded into FGENESH to recover the revised sequences of both transcript and protein (Figures S3 and S4). All the new sequences generated were included in the successive analyses performed below.

The *FaWRKY* genes were named following three criteria: foremost, due to their homology and shared synteny with the *FvWRKYs* (*FaWRKY1* to *FaWRKY64*), derived from the SynMap2 analysis; then by a letter indicating the subgenome donor (A, *F. nipponica*; B, *F. iinumae*; C, *F. viridis*; D, *F.vesca*); and finally, numbering the gene duplications, if any (.1, .2, etc.). The final list of the 257 *FaWRKY*s is provided in Table S3. Biochemical properties and the subcellular location of the FaWRKY proteins were also calculated and as for their FvWRKY orthologs, GRAVY values indicate that FaWRKYs are hydrophilic and more likely globular-shaped, and located in the nucleus, with few exceptions. Overall, the FaWRKY family shares a high gene structural conservation (Figure S5) and nucleotide sequence identity with their *FvWRKY* orthologs, with an average of 97.43%. Moreover, FaWRKY proteins were grouped according to their WDs, which match with their respective FvWRKY orthologs. Furthermore, this similarity includes the additional motifs found in many cases. Many WRKY core motif variations, some of them not present in the FvWRKY proteins, as well as truncated Znf motifs, domain loss and presence of additional duplicate motifs also found in the diploid, were detected. The number of WD modifications detected in the FaWRKY proteins is higher than the one found in the FvWRKYs, affecting FaWRKYs belonging to diverse groups, including the R protein-WRKY homologs. In FaWRKY51A.2, only one incomplete WD was found, lacking the Znf portion, and thus was not classified within any WRKY group. Interestingly, FaWRKY26B.2 is a chimeric protein harboring an additional Myb-like DNA-binding domain as well as two unusual, modified WDs (I-N and I-C).

### 2.2. Genome-Wide Distribution and Gene Duplications of the Strawberry WRKY Family

Duplicated genes are abundant in plant genomes. These duplicates are retained after whole-genome duplication (WGD) events and have contributed to the evolution of novel functions in plants [41]. Ancient or recent WGD and polyploidy are common among the angiosperms [42,43]. The homologous duplicated genes within the same genome are named paralogs, while the term homoeologs refers to the homologous genes resulting from allopolyploidy [43]. We have used DAGchainer [44], implemented in the SynMap2 web-based tool [45], in order to identify duplicated (paralogous) *FvWRKY* genes, as well as *FaWRKY* paralogous and homoeologous genes.

**Figure 2:**
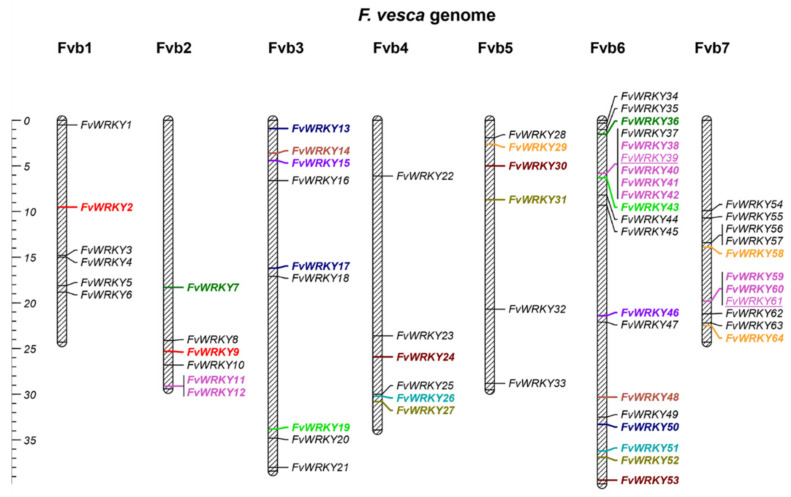
Chromosome mapping of the *Fragaria vesca WRKY* gene family. Segmental duplicated gene pairs (syntenic paralogs) share the same colors. Tandemly duplicated gene clusters are colored in pink, and underlined genes are collinear with genes outside that syntenic block. Ruler size is in Megabases.

**Figure 3:**
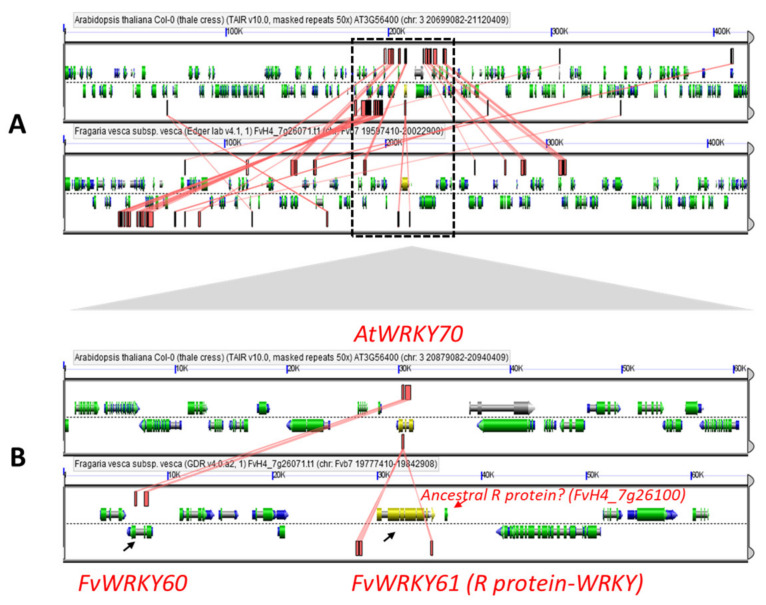
GEvo analyses comparing microsynteny between genomic regions from *A. thaliana* and *F. vesca*. (**A**) Genomic regions containing the genes *FvWRKY60*, *FvWRKY61* and *AtWRKY70* with red blocks and connectors show high-scoring sequence pairs between both sequences. The region shown in B is framed. (**B**) Homology details among *FvWRKY60*, *FvWRKY61* (black arrows) and *AtWRKY70*. A short, non-*WRKY* gene (*FvH4_7g26100*, red arrow) was identified as part of the *FvWRKY59-60-61* tandem (see text for details).

The *FvWRKY* genes are unevenly distributed among the seven chromosomes, with almost half of them (31 out of 64) located on chromosomes 6 and 7 (Figure 2). This is partly due to gene expansion by tandem and segmental duplications. Remarkably, 14 out of 15 group III *WRKY* coding genes are located on chromosomes 6 and 7. Sixteen segmental chromosome duplications and three groups of tandem repeats containing *FvWRKY* paralogous genes were found (Table 1). All the *FvWRKY* paralogs exhibit low ω values, indicating that they are under strong negative (purifying) selection pressure as observed for other species [46]. Duplicate gene pairs *FvWRKY13-50* and *FvWRKY17-50*; *FvWRKY24-53* and *FvWRKY24-30*; and *FvWRKY29-58* and *FvWRKY58-64* share a common gene, suggesting that they have evolved as a result of a two-step duplication event [47]. Interestingly, the two tandemly duplicated groups consisting of *FvWRKY38-39-40-41-42* and *FvWRKY59-60-61* seem to be related to each other. The *FvWRKY61* gene, belonging to the FvRWRKY subclass, was identified as a segmental duplicate of *FvWRKY39* within a tandem repeat along with *FvWRKY59* and -*60*, pinpointing the origin of this chimeric gene as a likely result of a genetic rearrangement of a group III *WRKY* gene and an unknown R gene leading to the formation of a novel R protein-*WRKY* gene. Actually, another non-*WRKY* gene (*FvH4_7g26100*) was identified as part of the *FvWRKY59-60-61* tandem, sharing partial sequence homology with *FvWRKY61*, as well as with a TIR-NB-LRR gene (*FvH4_7g17700*), suggesting a link with the origin of the R protein-like domain found in FvWRKY61 (Figure 3). Tandem or segmental gene duplications involving the other three members of the *FvRWRKY* group (*FvWRKY35*, -*55* and -*62*) were not detected, probably because duplication-inherent mechanisms, such as inversions or post-duplication events, have broken the collinear relationships among *FvWRKY* and the “donor” R genes [42]. However, a candidate process already proposed for the formation and posterior expansion of the R-WRKY protein class, besides WGD events, is gene transposition [22]. TE elements provide the capability to generate new genes by duplicating and recombining gene fragments [48]. In a recent study about the diversification of plant immune receptors, the authors concluded that the integration of exogenous domains into nucleotide-binding leucine-rich repeat (NLR) proteins combine both gene duplication and interchromosomal translocation pointing to TE elements or ectopic recombination as the most likely mechanisms [49]. Such events may also have led to the emergence of the R-WRKY protein families, independently, in several species.

**Figure 4:**
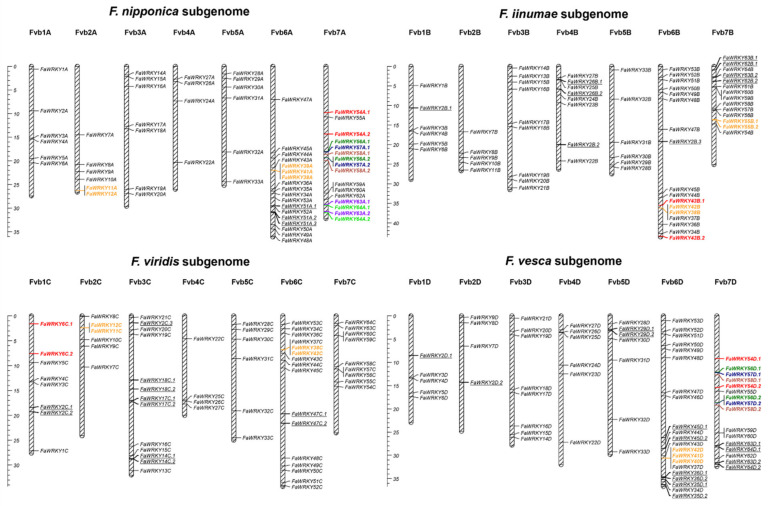
Chromosome mapping of the *Fragaria* × *ananassa WRKY* gene family within each subgenome. Segmental duplicated gene pairs (syntenic paralogs) share the same colors. Non-syntenic gene duplicates are underlined. Tandemly duplicated gene clusters are colored in orange. Ruler size is in Megabases.

The octoploid Fa genome is composed of four subgenomes (A, B, C and D) highly collinear with *F. vesca*, and it contains sets of homoeologous *WRKY* genes derived from the diploid ancestors (Figure 4). Genomic reorganizations and fractionation resulting from polyploidization [26,30] have caused some *FaWRKY* genes loss, as well as some segmental transpositions and inversions, creating collinearity breaks and hampering the identification of similar paralogous pairs as those found in the diploid. Instead, several new segmental gene duplicates were detected, as well as several non-syntenic gene duplications and triplications (Table 2). Ks values calculated for the octoploid paralogs are quite low in most cases, evidencing that gene duplications are very recent. Several *FaWRKY* paralogs show ω values greater than 1, which means that these genes may be undergoing positive selection, and sub-functionalization or neo-functionalization processes might be taking place [50]. Taken together, we could hypothesize that they have appeared as a result of either homoeologous exchanges, intrinsic to polyploidization [30], or post duplication events affecting single genomic features as gene transposition [42]. Indeed, TEs can be activated by polyploidy and hybridization events [51] and play important roles in producing segmental duplications in plants and in generating changes in the genome organization and size of the hybrids, which often produce synteny breaks [52,53,54].

### 2.3. Phylogenomic Analysis of the Strawberry WRKY Family

**Figure 5:**
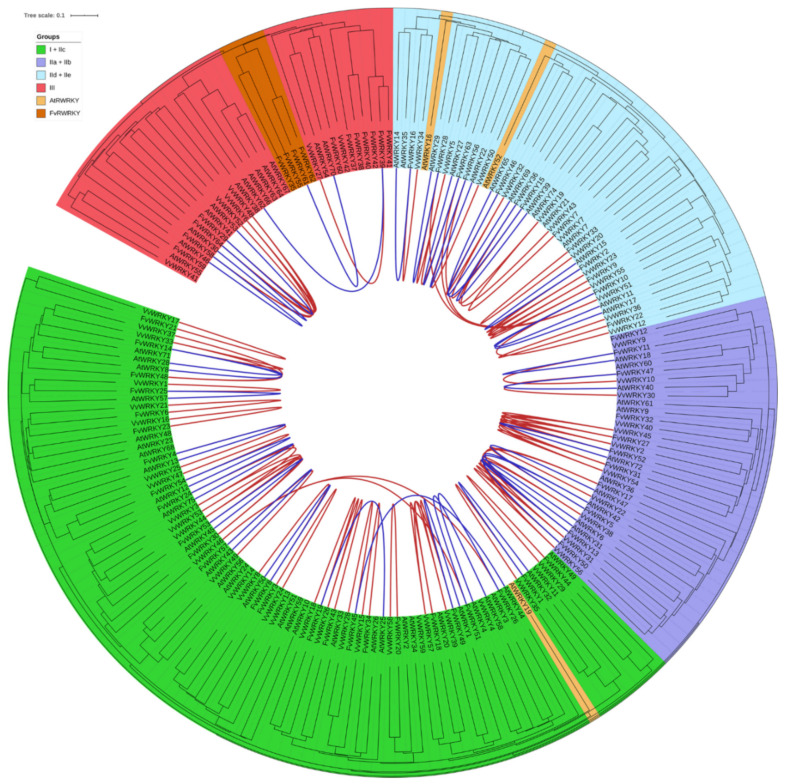
Phylogenetic analysis of *Fragaria vesca* (Fv), *Arabidopsis thaliana* (At) and *Vitis vinifera* (Vv) WRKY proteins. WRKY proteins are clustered into Groups I + IIc, IIa + IIb, IId + IIe and III. R protein-WRKY from Fv and At are clustered within their respective groups. The tree was inferred using the Neighbor-Joining method (1000 bootstrap replicates) and drawn to scale, with branch lengths in the same units as those of the evolutionary distances used to infer the phylogenetic tree. The evolutionary distances were computed using the p-distance method and are in the units of the number of amino acid differences per site. All positions with less than 95% site coverage were eliminated. Connecting lines represent shared synteny between Fv-Vv (red) and Fv-At (blue).

We have used a phylogenomic approach to study the evolution of the strawberry WRKY family and explore its potential biological implications. Shared synteny among *Fragaria vesca* (Fv), *Arabidopsis thaliana* (At) and *Vitis vinifera* (Vv) *WRKY* families was investigated using the CoGe web. SynMap3D was used to find collinear *WRKY* genes shared among the three species (Table S4). In addition, SynMap2 was used to find the collinear *WRKY* genes shared between Fv-At and Fv-Vv and not found by the SynMap3D analysis (Table S5). Full-length WRKY proteins from the three species were aligned by MUSCLE, and an unrooted phylogenetic tree was constructed and annotated using iTol to integrate both phylogenetic and collinearity relationships (Figure 5). The resulting tree distribution obtained meets the phylogenetic classification of the WRKY proteins into Groups I + IIc, IIa + IIb, IId + IIe and III [22].

Most of the *FvWRKY* genes share synteny with *AtWRKY* and *VvWRKY* members of the corresponding groups. Exceptions include the R protein-WRKY members, absent in Vv, and because At and Fv members harboring similar R domains also carry different WDs as previously described by [22]. The analysis of the ω values obtained for the syntenic At, Vv and Fv*WRKY* genes indicates that purifying selection is likely the main evolutionary driving force for this family in Rosaceae, acting against changes within the protein sequences, which modify or disrupt their functionality [55]. A higher number of collinear relationships are found with *VvWRKY* than *AtWRKY* genes, showing lower Ks values overall. This is probably because, unlike At, Vv has a relatively slow evolutionary rate [56,57]. Moreover, like Vv, the latest large gene expansion in the *Fragaria* lineage, likely took place in the gamma (γ) whole-genome triplication, shared by the core eudicots, since there is no evidence of more recent WGD events [28,56,58].

Synteny and collinearity conservation between *FvWRKY* and *FaWRKY* orthologs was also investigated with SynMap2, which found that most of the strawberry WRKYs were located within conserved blocks. Despite the fact that genomic reorganizations are widespread in the Fa genome relative to the diploid, 189 of 257 *FaWRKY* genes are syntenic and collinear with their *FvWRKY* orthologs, regardless of their subgenome origin (Table S6). A phylogenetic tree of the diploid and octoploid strawberry WRKY proteins was constructed and annotated with the shared synteny information (Figure S5). The tree distribution obtained for the strawberry full WRKY proteins is also according to the phylogenetic classification of the WRKYs into Groups I + IIc, IIa + IIb, IId + IIe and III.

The ω values show that strawberry WRKY orthologs are predominantly under purifying selection, indicating high conservation of the *WRKY* gene family within the two *Fragaria* species. However, some cases of positive and possible neutral selection (due to very low Ks values and thus considered as no evolution) are also uncovered, indicating that some genes may be undergoing neo or subfunctionalization processes. Interestingly, most of the synteny and collinearity breaks between the diploid and octoploid species are observed in those *WRKY* genes located in chromosomes 6 and 7 (52 out of 68), which appear to have undergone substantial genomic rearrangements in Fa, such as fractionation (loss of genomic features, such as genes) and ectopic recombination with non-homologous chromosomes (Figure S6). Thus, extensive homoeologous gene losses were detected for *FaWRKY39*, -*40,* -*46* and -*61* genes, which retain only one of the homoeologs. Several members of *FaWRKY* group III are also affected by homoeologous gene losses, perhaps because most of this group is located within these two chromosomes.

### 2.4. Orthology Relationships and Annotation of the Strawberry WRKYs

Orthologs are pairs of genes found in different species and originated from a speciation event [43]. There is a widespread belief that orthologs are more prone to conserve similar functions than paralogs [59]. This concept, known as the “ortholog conjecture”, is the basis for predicting conserved gene functions. Moreover, syntenic conservation between orthologous genes is less likely to undergo positive selection and, therefore, more likely to retain a conserved function [43,60]. However, inferring orthologs can be challenging, depending on the evolutionary distances and the occurrence of WGD events that produces gene duplication/loss.

**Figure 6:**
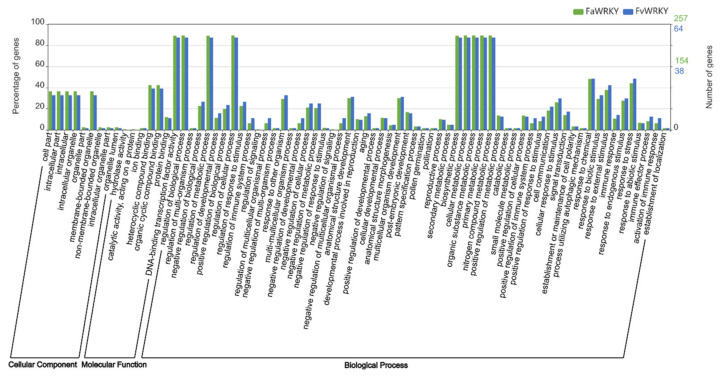
GO functional annotation plot comparing the number and percentage of FvWRKY and FaWRKY proteins sharing the same functions. The *y*-axis represents the number (right) and the percentage (left) of WRKY genes in *Fragaria* × *ananassa* (green) and in *Fragaria vesca* (blue) for each GO category (*x*-axis).

Orthologs of Fv and Fa WRKY proteins were investigated using the new eggNOG 5.0 Database and hierarchically classified into orthologous groups (OGs) within several taxonomic scopes (Table S7). Consistent with the high sequence conservation detected by the previous analyses, the putative FvWRKY and FaWRKY orthologs were classified into the same OGs. Moreover, GO functional annotations are essentially shared by both species (Figure 6). The only exceptions were FaWRKY37D and the chimeric protein FaWRKY26B.2, which were assigned to different OGs than their respective orthologs and homoeologs. Moreover, FaWRKY37D was classified into OGs containing non WRKY proteins of unknown function, such as AT2G38570.1. Candidate protein orthologs in several plant species can be identified in the eggNOG 5.0 Database using this hierarchical OG classification. However, we concentrated our attention on the homology with the AtWRKY family, which has been the best functionally characterized so far. The taxonomic scope used was the closest level to fabids (clade including the Rosaceae, to which belongs the genus *Fragaria*) [61] containing orthologous AtWRKY proteins. Thus, up to 17 one-to-one AtWRKY orthologs were identified, but one-to-many or many-to-many AtWRKY orthologs with the strawberry WRKY family were also found.

**Figure 7:**
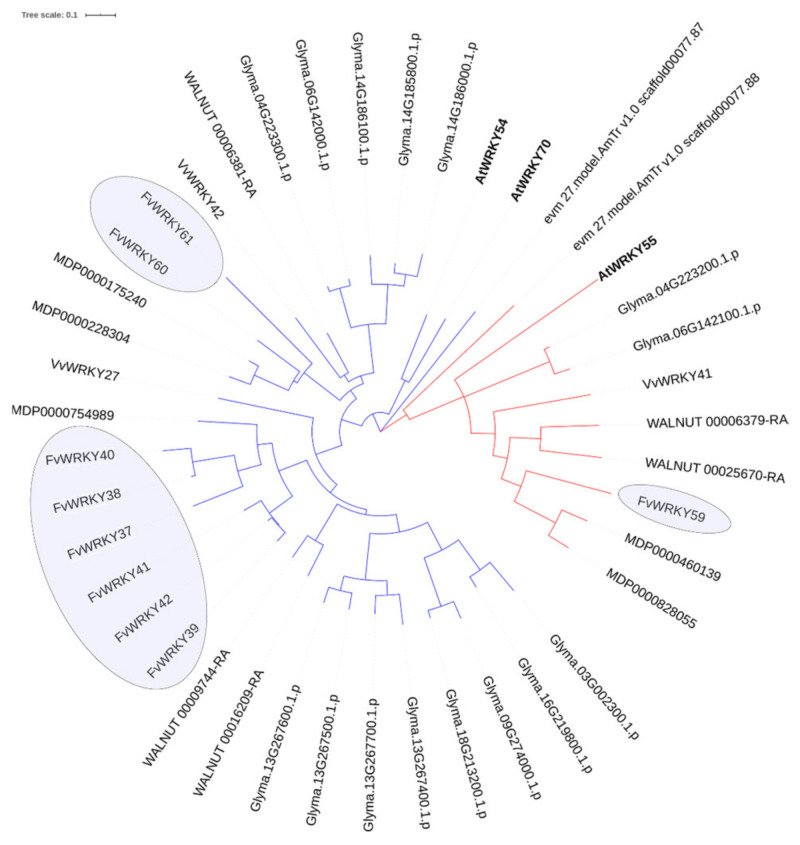
Phylogenetic tree of the *AtWRKY55* and *AtWRKY54/70* orthologous genes (red and blue branches, respectively) in strawberry (Fv), soybean (Glyma), walnut (WALNUT), apple (MD), grapevine (Vv) and *Amborella* (*AmTr*). Strawberry orthologs to *AtWRKY54/70* paralogs underwent tandem duplications after the gamma hexaploidization and independently to the more recent WGD events in other species. The evolutionary history was inferred using the Maximum Likelihood method (100 bootstraps) with optimized parameters (TN + G + I). The tree is drawn to scale, with lengths of branches representing the number of substitutions per site. Analyses were conducted in MEGA7.

For example, several FvWRKY members (FvWRKY37, -38, -39, -40, -41, -42 and -60) were found homologous to the *Arabidopsis* WRKY54/70 proteins. The phylogenetic reconstruction using all the potential eggNOG homolog genes in several species (Table S8) by the maximum likelihood method shows that the strawberry *AtWRKY54/70*-like orthologous genes were originated by different rounds of gene duplication (Figure 7). Thus, the strawberry orthologs of the two Vv gamma-paralogs *VvWRKY27* and *VvWRKY42* experienced successive tandem duplications, originating the *FvWRKY37-38-40*, *FvWRKY39-41-42* and *FvWRKY60-61* tandem gene groups. Independently, the *AtWRKY54-70* gene duplication likely took place in the more recent At-β or At-α [62], as well as other WGD events in *Juglans*, *Malus* and *Glycine* expanded their WRKY orthologs [63,64,65,66]. Therefore, it is likely that several strawberry WRKY genes, homologous to *AtWRKY54-70*, share some ancestral functions of the last ones, which still today mainly show redundant roles [6,67,68]. The two *Amborella trichopoda* orthologs included help to show that divergence between the *AtWRKY55*-like and *AtWRKY54/70*-like genes probably predates the gamma event since it is not shared by *Amborella* [69]. This also would explain the gene *FvWRKY59*, identified as tandem duplication along with *FvWRKY60-61*, as an ancient pre-gamma paralog within this tandemly duplicated group.

### 2.5. Expression Analysis of FvWRKY Genes in Different Tissues, Development Stages and Growth Conditions

Most of the woodland strawberry WRKYs have not been yet functionally characterized nor fully described at the expression level, with few exceptions [70,71,72]. Consequently, to gain insight into their possible biological roles, the expression pattern of the complete *FvWRKY* family was analyzed in several tissues (Figure S7), considering the transcript expression values collected from previous research [29] (Table S9).

The analysis revealed that four genes, *FvWRKY10*, -*2*, -*7* and -*33*, exhibit high transcript accumulation in all samples except pollen, suggesting that they should play important roles in most phases of strawberry physiology. Both transcript abundance and expression patterns of the remaining *FvWRKY* genes vary largely in different vegetative and reproductive tissues, as well as during receptacle ripening and pathogen challenge. For example, several *FvWRKY* genes changed their transcripts abundance or expression profiles during ripening in receptacle from two botanical varieties of *F. vesca*, Ruegen (F7–4) and Yellow Wonder 5AF7, which produce fruits with red or yellow flesh and skin, respectively [73,74]. However, the changes detected in the expression pattern from 15 days to 22 days post-anthesis for *FvWRKY2*, -*23*, -*29*, -*36*, -*47*, -*50* and -*51* were similar in both accessions, pointing out that these genes may play key roles in the strawberry fruit ripening process.

A greater number of *FvWRKY* genes (49 out of 64) changed their expression pattern in roots infected by the hemibiotrophic oomycete *Phytophthora cactorum*, with transcript abundance differences that reflect both positive and negative regulation in response to this pathogen [75]. Notorious up-regulation was detected for collinear and orthologous genes of well-known *AtWRKY* TFs involved in plant defense responses (see Tables S4, S5 and S7), as FvWRKY3 and -8, homologous to AtWRKY50/51, mediators of the SA and JA signaling [76,77]; FvWRKY24, -30 and -53, orthologs of AtWRKY45 and -75; AtWRKY75, and the *F. ananassa* ortholog FaWRKY24 (previously reported as FaWRKY1) have been described as positive and negative regulators of plant defense in *Arabidopsis* and *F. ananassa*, respectively [78,79,80]; FvWRKY29, ortholog of AtWRKY53, which positively modulate SAR in *Arabidopsis* [81,82]; FvWRKY38, -42 and -60 share homology with AtWRKY54 and -70, its closest homologs, which seem to regulate the balance between SA and JA-dependent defense pathways, acting as a negative regulator of SA biosynthesis [68]; FvWRKY19, -43 and -47 that share orthologous group with AtWRKY25, AtWRKY33 and AtWRKY18/40, respectively, major factors predicted to function as important hubs within the WRKY network of plant defense in *Arabidopsis* [83,84]. Promoter analysis of these genes show an abundance of predicted cis-acting elements in response to methyl-jasmonate in *FvWRKY3*, -*8*, -*24*, -*29*, -*38*, -*42*, -*43*, -*47*, -*53* and -*60*. Moreover, *FvWRKY43* and -*47* (*AtWRKY33* and *AtWRKY18/40* orthologs, respectively) contain salicylic-responsive elements. All these cis-acting elements (MeJa and SA response elements) seem to be conserved in their *F. ananassa* homoeologs (Tables S10 and S11). All in all, these data suggest an outstanding involvement of the WRKY TF family in the woodland strawberry defense response.

**Figure 8:**
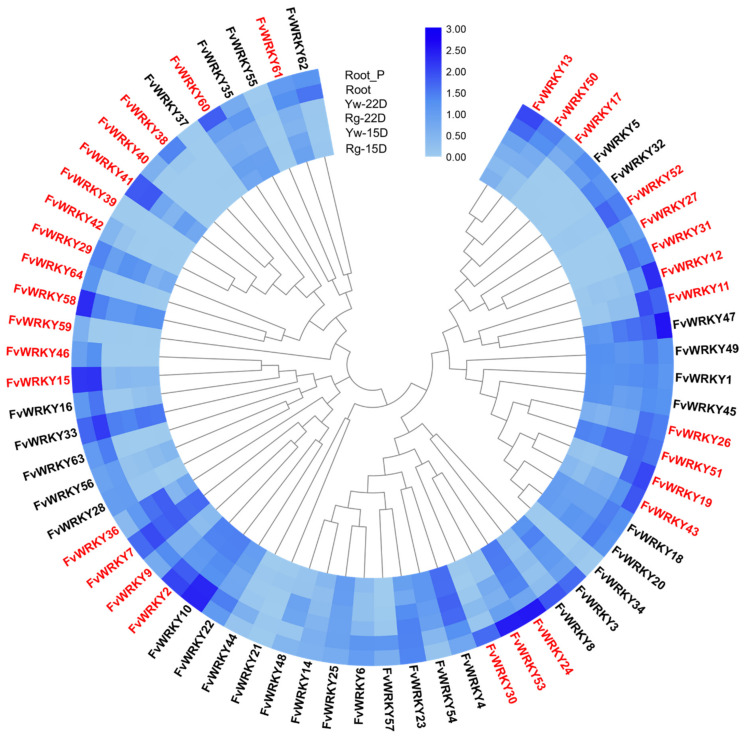
Phylogenetic tree of the *FvWRKY* family showing expression values during fruit ripening (Ruegen receptacle tissue at 15 and at 22 DPA and Yellow Wonder receptacle tissue at 15 and at 22 DPA: Yw-15D, Yw-22D, Rg-15D and Rg-22D, respectively) and roots (Root, collected from 7-week-old plants grown in aerated hydroponic culture, and Root_P, after 2 days of inoculation with *Phytophthora cactorum*). *FvWRKY* paralogs are shown in red (see Table 1. Color scale represents the expression level as log-transformed TPM (Transcripts per Million) values.

A closer look at the transcription pattern (Figure 8) reveals substantial differences in the transcription level of most paralogous pairs, as well as in their expression profiles, which suggest that they are not fully redundant and may have undergone functional divergence, neo or sub-functionalization [85]. Thus, duplicate genes *FvWRKY2-9*, *FvWRKY7-36*, *FvWRKY13-50*, *FvWRKY17-50*, *FvWRKY19-43*, *FvWRKY24-30*, *FvWRKY24-53*, *FvWRKY29-58* and *FvWRKY58-64* in receptacle ripening; *FvWRKY2-9*, *FvWRKY11-12* and *FvWRKY15-46* in root infection; and members of the tandem duplications *FvWRKY38-39-40-41-42* and *FvWRKY59-60-61* in both receptacle ripening and root infection, show very different expression patterns.

### 2.6. Expression Analysis of the FaWRKY Gene Family in Strawberry Tissues, the Fruit Ripening Process and Defense Responses

The recent publication of a high-quality annotated octoploid strawberry genome, and the availability of public RNA-seq data, provide a valuable opportunity to investigate the expression patterns of the *FaWRKY* gene family in different tissues and growth conditions at the level of homoeologs and paralogs to understand their functions in key strawberry processes, such as fruit ripening and defense responses against pathogens. Thus, we have investigated two publicly available strawberry datasets, featuring both tissue expression profiles and transcriptomic changes along four stages of achene and receptacle ripening [35] and anthracnose defense response of strawberry leaves infected with the hemibiotrophic fungus *Colletotrichum fructicola* [86]. Both raw and differential gene expression values are provided in Tables S12 and S13, respectively.

The expression pattern for most of the *FaWRKY* genes varied among the different strawberry tissues investigated, showing many cases of preferential or even tissue-specific expression (Figure S8 and Tables S13–S15). For example, *FaWRKY17B/17C.1/17C.2/17D* were detected and differentially expressed exclusively in roots, whereas the homoeolog *FaWRKY17A* was expressed in roots, leaves and green receptacles. On the other hand, *FaWRKY24A/24B/24D*, among others, showed a higher transcript accumulation in roots than in any other tissues. The strawberry *FaWRKY24* homoeologs were identified as orthologs of AtWRKY75, which acts as a negative regulator of root development as well as a positive regulator of Pi stress responses [87].

**Figure 9:**
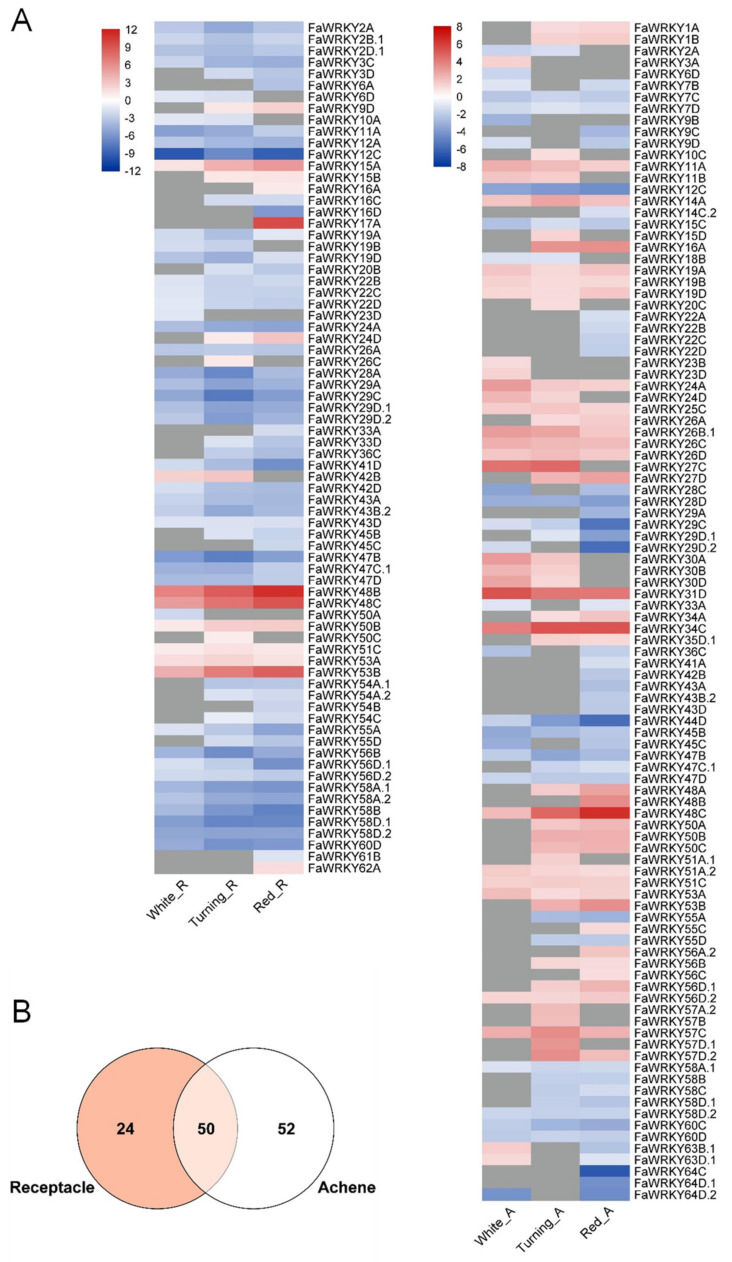
(**A**) Heatmap of differentially expressed *FaWRKY* genes during strawberry fruit ripening stages in receptacle (White_R, Turning_R, and Red_R; left side) and achene (White_A, Turning_A, and Red_A; right side) tissues. Changes in gene expression, with respect to green fruit tissues, are represented as log2 fold change if absolute values were >1 (padj < 0.01), otherwise, they were colored in grey. (**B**) Venn diagram showing those genes which were differentially expressed in receptacle and achene only, or in both.

Fruit ripening is a critical developmental process in strawberry, and fruit size, color and aroma, are important agronomical traits. However, little is known about the involvement of members of the strawberry *WRKY* gene family in the genetic control of ripening and the major changes undergone in strawberry fruit. Nevertheless, transcriptional reprogramming of *FaWRKY* genes is evident in both receptacle and achene from immature (green) to mature (red) fruit (Figure S10; Tables S16 and S17). We have found that 74 *FaWRKYs* were significantly up or down-regulated in receptacles during specific stages of ripening, while 102 were in their respective achenes (Figure 9A). Of these, 24 and 52 *FaWRKY* genes were differentially expressed only in receptacles or achenes, respectively (Figure 9B). Moreover, this analysis revealed that many paralogous and homoeologous genes were expressed differently in both fruit tissues (Table S18), suggesting that they may have undergone functional divergences in the strawberry fruit ripening.

It has been shown that the hormonal balance between abscisic acid (ABA) and auxins is primarily responsible for the changes leading to ripening in this non-climacteric fruit [34]. Supporting this, the expression in the receptacle of three strawberry *WRKYs* (Fv *gene28720*, *gene19478* and *gene01340*) have previously been described to be activated by ABA and repressed by auxins in the receptacle, while another three WRKY genes (Fv *gene07210*, *gene03411* and *gene09147*) were repressed by auxins and not affected by ABA [34]. Our transcriptomic analysis supports potential roles for five of these genes at some points along the strawberry ripening process. Thus, genes *FaWRKY48B/48C* (Fv *gene28720*) and *FaWRKY53A/53B* (Fv *gene01340*) were differentially up-regulated in both receptacle and achene, whereas the homoeologous *FaWRKY48A* was only in achene. On the other hand, *FaWRKY24D* (Fv *gene07210*) was differentially up-regulated in both receptacle and achene, but its homoeologous *FaWRKY24A* was down-regulated in receptacle and up-regulated in achene. Further, genes *FaWRKY17A* (Fv *gene03411*) and *FaWRKY9D* (Fv *gene09147*) were up-regulated in red receptacle, whereas *FaWRKY9B/9C/9D* homoeologs were down-regulated in achene. However, none of the *FaWRKY13* homoeologs (Fv *gene19478*) were differentially expressed. Interestingly, ABRE cis-acting elements are abundant in the promoter regions of genes *FaWRKY48A/48B/48C*, *FaWRKY53A/53B*, *FaWRKY24A/24D* and *FaWRKY17A*, while auxin-responsive elements are present to a lesser extent (Table S10), which seems to be conserved from their FvWRKY orthologs (Table S11).

To obtain further insight into the potential roles of the differentially expressed *FaWRKY* genes during fruit ripening, we constructed a functional protein association network in STRING using the experimentally known interactions for their Arabidopsis orthologs (Figure S11). The resulting network connects FaWRKY24, FaWRKY57 and FaWRKY47 with clusters of proteins related to the proteasome and developmental processes such as photomorphogenesis and responses to auxin and jasmonic acid. On the other hand, FaWRKY43 and FaWRKY47 (both significantly down-regulated during fruit ripening) interact with proteins involved in the biotic and abiotic stress responses. Several strawberry VQ proteins, which change their expression profiles during fruit ripening [88], appear as interacting nodes with these FaWRKYs and could modulate their transcriptional activity. Finally, FaWRKY11 and FaWRKY47 are associated with members of the bHLH transcription factor family, involved in the regulation of multiple processes such as anthocyanin biosynthesis and cell elongation during strawberry fruit ripening [89,90]. These findings evidence that some WRKY TFs could play important roles in the strawberry fruit ripening and further research is needed to unravel their specific function.

**Figure 10:**
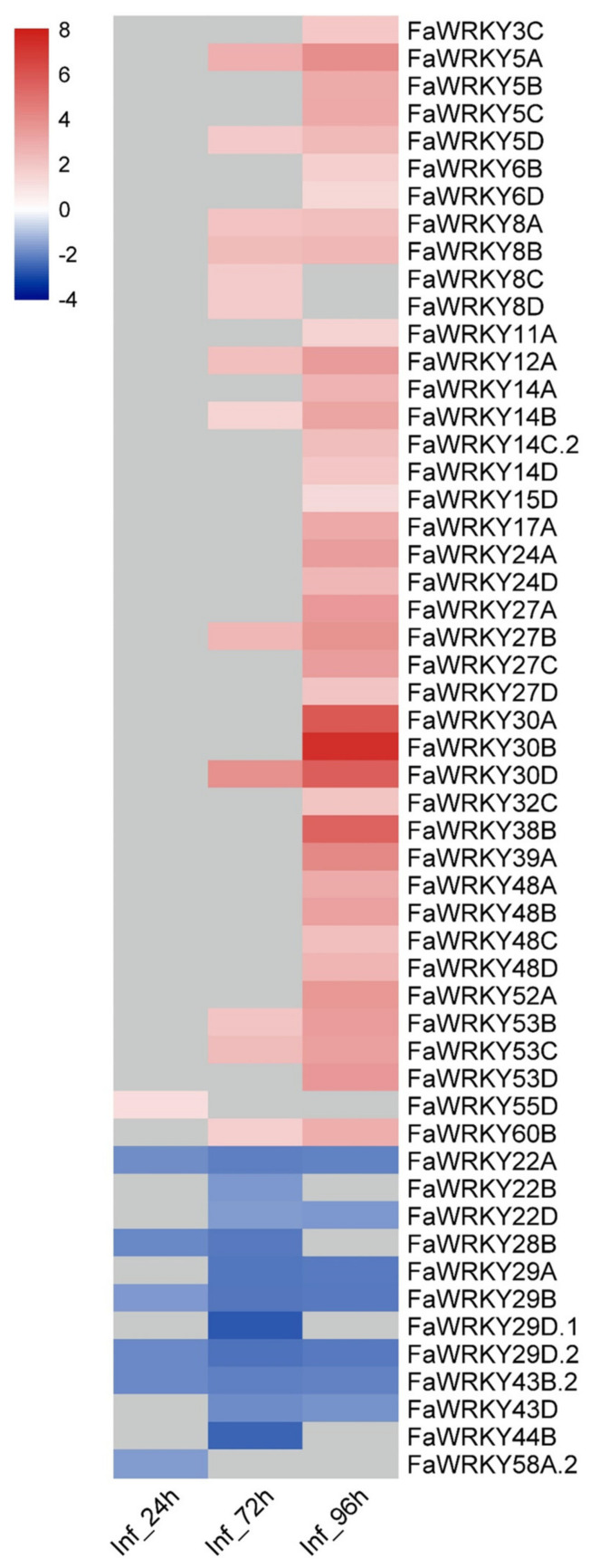
Heatmap of differentially expressed *FaWRKY* genes in response to *C. fructicola* infection in leaves. Changes of gene expression in infected strawberry (Inf_24h, Inf_72h, Inf_96h; 24, 72 and 96 h after spore inoculation, respectively) vs. mock-inoculated strawberry are represented as log2 fold change if absolute values were >1 (padj < 0.01), in a color scale from lowest (blue) to highest (red). Otherwise, they were colored grey.

It is known that WRKY TFs have important roles in defense responses against pathogenic fungi [20]. The expression patterns of several *FaWRKY* genes fluctuated in strawberry leaves challenged with *C. fructicola* (Figure S12 and Table S17). A total of 53 *FaWRKYs* were differentially expressed, 41 were up-regulated and 12 were down-regulated (Figure 10), most of which were homologs of well-known defense-related WRKYs. Among the *FaWRKY* up-regulated genes with higher expression and having known functions in plant defense, we found WRKY8/28-probable orthologs *FaWRKY14A*, -*14B*, -*14C.2* and -*14D*; WRKY54/70 orthologs *FaWRKY38B*, -*39A* and -*60B*; WRKY75 orthologs *FaWRKY24A* and -*24D*; WRKY50/51 orthologs *FaWRKY3C*, -*8A*, -*8B*, -*8C* and -*8D*; WRKY72 ortholog -*52A*; and WRKY18 ortholog *FaWRKY11A*. On the other hand, among the down-regulated *FaWRKY* genes were the *WRKY53* orthologs *FaWRKY29A*, -*29B*, -*29D.1* and -*29D.2.* Several of these genes are involved in the negative regulation of JA-mediated, but positive regulation of SA-mediated defense responses [77,91,92,93,94,95], while WRKY18 positively regulates the expression of some key JA-signaling genes in *Arabidopsis* [96,97]. It is worth mentioning that AtWRKY33 high-homology-sharing genes *FaWRKY43B.2* and *FaWRKY43D* are down-regulated along the time course of infection. *Arabidopsis* WRKY33 is a key positive regulator of the JA-mediated defense response against necrotrophic fungi, also showing an antagonistic effect with the SA-mediated pathway [98], and AtWRKY33 orthologs seem to share similar functions in other plant species, including the woodland strawberry [71]. Although this down-regulation was already noticed by Zhang et al. [86], it contrasts markedly with the results reported in other strawberry plant tissues of either resistant or susceptible cultivars challenged with *Colletotrichum gloeosporioides* [99] or *Colletotrichum acutatum* [37,88]. However, it could be explained if subtle differences in the pathogen-specific strategies do exist among *Colletotrichum* species to manipulate the host defense in different strawberry tissues.

Homoeologs of *FaWRKY24* and *FaWRKY53* are both AtWRKY75-like factors and, as mentioned before, FaWRKY24 (formerly, FaWRKY1) and AtWRKY75 have been previously reported to play important roles as positive regulators of plant defense in *A. thaliana* against *Pseudomonas syringae* [37,79]. Both the Fv paralogs *FvWRKY24* and *FvWRKY53* and their Fa orthologs *FaWRKY24A/24D* and *FaWRKY53B/53C/53D* were also detected to be up-regulated in response to different pathogens. In addition, recent research has shown that WRKY75 is a positive regulator of the JA-mediated defense against the necrotrophic fungi *Botrytis cinerea* and *Alternaria brassicicola* [78]. Interestingly, *FaWRKY24* (*FaWRKY1*) homoeologs are also up-regulated during strawberry fruit ripening as well, as they have been reported as negative regulators of the resistance to *C. acutatum* in strawberry fruit [80]. This illustrates the dual roles that the strawberry AtWRKY75-like factors may play against different pathogens or in different plant tissues.

Among the R protein-WRKY subclass, FaWRKY55D was the only one differentially expressed at early stages of the infection. This is particularly interesting as it could be hypothesized that FaWRKY55D could participate in the activation of the early immune response in strawberry similarly to the RRS1-R/RSP4 pair system (via an integrated WRKY domain in RRS1-R) recently described in Arabidopsis [100,101]. Therefore, it should be included in the repertory of putative candidate strawberry *WRKY* defense-responsive genes for further study. Several homoeologs of the other *FaRWRKY* genes (*FaWRKY35*, *FaWRKY61* and *FaWRKY62*) were expressed in both mock-treated and infected tissues. However, their transcript abundances did not change significantly in the course of infection. Most of them showed relatively high transcript abundances in leaves, roots and fruit tissues also, suggesting that changes in their gene expression, if any, are either not needed to play positive roles against pathogens or these changes are pathogen-specific deployed or involved in other plant processes. Nevertheless, their roles in the strawberry physiology remain unknown, as the vast majority of other R protein-WRKY in several species [100,101].

From the currently available data, a selection of putative candidate *FaWRKY* genes for further research is shown in Table S19. This set of outstanding genes was selected based on the results shown in the heatmap of *FaWRKY* genes differentially expressed during strawberry fruit ripening (Figure 9) and in response to *C. frutícola* infection (Figure 10), and also taking into consideration the abundance of predicted cis-acting elements within the promoter region of each member of the *FaWRKY* family (Table S10).

## 3. Conclusions

The present study represents a novel and comprehensive update of the strawberry WRKY family using the ultimate and most accurate genome assemblies and gene annotations available. The use of the most recent data has allowed us to perform more precise analyses and characterization of the *WRKY* gene family composition, its evolutionary history and gene expression patterns. In the case of the diploid *F. vesca*, we have made extensive new findings not covered by previous works. As for the cultivated octoploid hybrid *Fragaria* × *ananassa*, we have described for the first time the *WRKY* homoeologs and gene duplications. We have reanalyzed public data to study their expression in several tissues, potential roles in defense and fruit ripening and discussed the results in the light of the precise identification of the strawberry WRKY family, particularly in the case of *Fragaria* × *ananassa*.

Thus, a total of 64 *WRKY* genes are present in the genome of the diploid *Fragaria vesca* and 257 corresponding *WRKY* orthologs in the genome of the cultivated allo-octoploid *Fragaria* × *ananassa* (cv. Camarosa). Our results show that the strawberry WRKY family preserves a high degree of conservation between both wild-type and cultivated species. Synteny analysis showed that *FaWRKY* genes are largely syntenic and collinear with their *FvWRKY* orthologs, despite some gene loses and synteny breaks detected in the octoploid, probably as a result of genomic rearrangements derived from hybridization and polyploidy. The strawberry WRKY family has been expanded by ancient WGD events, originating from several segmental and tandem duplications resulting in several paralogous genes. Moreover, most *FaWRKY* paralogs are not mirrored in *F. vesca* and could be inherited from the parental octoploid species *F. virginiana* and *F. chiloensis*, in which polyploidization could originate synteny breaks among ancestral paralogs as well as new gene duplications.

Gene expression is related to functionality, and its analysis can shed light on the biological roles of the genes studied. Thus, the expression patterns of most *WRKY* genes differed in the strawberry tissues here considered, evidencing differences in functional relevance across different tissues and growth conditions. Importantly, many strawberry *WRKYs* remarkably changed their transcript level or were differentially expressed in defense and fruit ripening stages, indicating their importance in these important biological processes. It is worth noting genes *FaWRKY38B*, -*39A* and -*60B* (*AtWRKY54/70* orthologs), *FaWRKY24A/D* and -*43B.2/D* (*AtWRKY75* and -*33* orthologs, respectively), *FaWRKY30A/B/D* and *FaWRKY55D* (encoding an R-protein) are related to pathogen defense, and *FaWRKY9D*, -*17A*, -*48B/C*, -*53A/B* are related with fruit ripening. The genes *FaWRKY11* and *FaWRKY47*, which seem to be related to bHLH transcription factors, and *FaWRKY57* are related to members of the VQ protein family, are also relevant. In addition, differences in the expression pattern detected among several *FaWRKY* paralogs and homoeologs point out either a finely regulated gene expression strategy to achieve putative additive genetic effects or evolutionary functional divergences. These data can help future studies deepen our understanding of the strawberry WRKY TF’s regulatory roles. Future RT-qPCR analyses of expression profiles of candidate *WRKY* genes under different development and stress conditions (including stress conditions not described in this study, such as salt, heat and osmotic stress) should confirm and expand the data retrieved from publicly available RNA-seq data, as well as help new approaches in breeding programs.

## 4. Materials and Methods

### 4.1. Identification of WRKY Family Members

The sequences of *Fragaria vesca* (Fv; Genome Assembly v4.0.a1 & Annotation v4.0.a2) and *Fragaria* × *ananassa* cv. Camarosa (Fa; Genome Assembly v1.0 & Annotation v1.0.a1) were retrieved from The Genome Database for Rosaceae (GDR) website (https://www.rosaceae.org/, accessed on 14 April 2022) [102]. The Hidden Markov Model of the WD (PF03106) was downloaded from the Pfam database [103] and used as a query in HMMER3 search, performed in the freeware tool UGENE v1.21 with default settings [104]. The candidate sequences were further confirmed to include the WD, as well as additional protein domains, using the Conserved Domain Database (CDD) [105]. WRKY sequences from *Arabidopsis thaliana* (At) [1], *Vitis vinifera* (Vv) [106] and several species were retrieved from the Plant Transcription Factor Database (PlantTFDB) [107]. Chromosome maps of the strawberry WRKY genes were drawn with MapChart v2.32 [108]. Protein locations were predicted using LOCALIZER (https://localizer.csiro.au/, accessed on 14 April 2022) [109]. General sequence handling and intron-exon structure analysis were performed using TBTools v1.055 WRKY protein properties calculation was carried out in the Freiburg Galaxy server (https://usegalaxy.eu/, accessed on 14 April 2022) [110].

### 4.2. Phylogenomics

Full WRKY protein sequences were aligned by MUSCLE to generate unrooted phylogenetic trees by the Neighbor-Joining (N-J) method in MEGA 7.0 [111]. The resulting protein trees were annotated with iTOL (https://itol.embl.de/, accessed on 14 April 2022) [112]. The compared synteny among species and strawberry WRKY gene duplications were studied in the CoGE (https://genomevolution.org/coge/, accessed on 14 April 2022) web-platform using LAST to find gene homologies and SynMap2 or SynMap3D to find collinear blocks shared by two or three species, respectively (Table S20) [42,45]. The non-synonymous (Kn) and synonymous (Ks) substitution rates between pairs of syntenic genes were calculated by codeml, implemented in SynMap, or in PAL2NAL (http://www.bork.embl.de/pal2nal/, accessed on 14 April 2022) [113] for duplicated genes lacking syntenic conservation. The non-synonymous (Kn) and synonymous (Ks) substitutions were used to calculate the Kn/Ks ratios (ω) between paralogous strawberry WRKYs and thus estimate the selection pressure. Values of ω > 1 or ω < 1 indicate positive or purifying (negative) selection, respectively, while ω = 1 means neutral (absence of) evolution [114]. Very low substitution values (Ks < 0.01) were considered as the virtual absence of nucleotide mutation and thus not accounted to calculate ω because it may result in inaccurate estimates [115,116]. eggNOG-Mapper v2 (http://eggnog-mapper.embl.de/, accessed on 14 April 2022) and the eggNOG 5.0 Database (http://eggnog5.embl.de/#/app/home, accessed on 14 April 2022) [117,118] were used to classify the WRKY orthologs in strawberry and other species, including *Amborella trichopoda*, *Juglans regia*, *Malus domestica* and *Glycine max*. GO terms with experimental evidence were acquired from the eggNOG results and plotted using WEGO 2.0 (https://wego.genomics.cn/, accessed on 14 April 2022) [119]. The homology with Arabidopsis proteins was applied in the construction of a protein functional association network in STRING (https://string-db.org/, accessed on 14 April 2022) [120], filtering interactions by high confidence (0.700) and using as active interaction sources “Experiments” and “Co-expression”.

### 4.3. Identification of Cis-Acting Element Analysis of WRKY Genes

The 2000-bp sequence upstream from the start codon (ATG) of each *WRKY* gene was obtained from the *Fragaria* × *ananassa* cv Camarosa (Annotation v1.0.a1) and *Fragaria vesca* (Annotation v4.0.a2) genome database for Rosaceae (GDR) website (https://www.rosaceae.org/, accessed on 22 May 2022). These sequences were used to identify cis-acting regulatory elements with the online program PlantCARE (http://bioinformatics.psb.ugent.be/webtools/plantcare/html/, accessed on 22 May 2022, Ghent, Belgium). The analysis of the frequency of the different cis-acting elements in the promoter region of each *WRKY* gene was obtained by Panda Library (phyton) with Jupyter notebook.

### 4.4. Expression Analyses of Diploid and Octoploid Strawberry WRKYs

Expression data of *FvWRKY* genes were taken from a previously published RNA-seq expression analysis in several *F. vesca* tissues and developmental stages [29]. Transcripts per Million (TPM) values were log10-transformed and depicted using the heatmap function of TBTools. The expression patterns of the *FaWRKY* genes were obtained through a complete reanalysis of several *Fragaria* × *ananassa* RNAseq datasets, including strawberry plant and fruit tissues (3 biological replicates in 54 RNAseq libraries: six independent green receptacle/achene libraries, six white receptacle/achene libraries, six turning receptacle/achene libraries, six red receptacle/achene libraries, three root libraries and three leaf libraries) [35]; and strawberry leaves infected by *C. fructicola* (three biological replicates) [86]. Detailed experimental procedures can be found within the original studies. Therefore, raw reads from the sequencing platform were processed to retain only high-quality sequences to be subsequently used for the mapping (Cutadapt v1.9, BBDuk v35.43). Sequencing adapters were first clipped from each library, and low-quality bases were trimmed. A Phred quality score of 24 was selected as the threshold, and reads with a length less than 30 nt were filtered out. Reads quality assessment was carried out using FastQC software (v0.11.8) to evaluate the effect of every step of this process. All subsequent analyses were conducted using these high-quality datasets. The remaining ribosomal RNA was detected by SortMeRNA software (v2.1) [121]. Thus, adaptors clipped reads were mapped to SortMeRNA prepackaged databases id98 (from Silva v119 and Rfam) with default parameters. A two-pass mode mapping was carried out by STAR (v2.7.3.a) [122] with parameter “--quantMode GeneCounts” in the second pass to extract raw counts per annotated gene ID according to each particular library’s strands. The obtained expression matrix was then supplied to the R library Deseq2 (v1.28.1) for a differential expression analysis (padj < 0.01 and absolute value of log2 fold change > 1). In addition, hierarchical clustering and heatmap analysis were performed for those genes of interest. Quantification values were z-scored prior to the clustering analysis by Pearson correlation with method “complete” (hclust function in stats package from R). Additional figures were drawn using TBtools.

## Figures and Tables

**Table 1:** *FvWRKY* gene duplications. Synmap2 did not provide the nucleotide substitution values (Kn, Ks) nor ω for tandemly duplicated genes. Genes marked with asterisks are shared by different duplicate groups.

Gene 1	Gene 2	Gene 3	Gene 4	Gene 5	Kn	Ks	ω
**Segmental duplications**							
*FvWRKY2*	*FvWRKY9*				0.4583	6.7082	0.0683
*FvWRKY7*	*FvWRKY36*				0.3183	1.5265	0.2085
*FvWRKY13*	*FvWRKY50* *				0.3550	2.6663	0.1331
*FvWRKY14*	*FvWRKY48*				0.4566	2.6947	0.1694
*FvWRKY15*	*FvWRKY46*				0.4185	5.7462	0.0728
*FvWRKY17*	*FvWRKY50* *				0.3908	5.7974	0.0674
*FvWRKY19*	*FvWRKY43*				0.3680	1.8100	0.2033
*FvWRKY24* *	*FvWRKY53*				0.4702	3.1511	0.1492
*FvWRKY24* *	*FvWRKY30*				0.4020	5.0096	0.0802
*FvWRKY26*	*FvWRKY51*				0.3295	3.0282	0.1088
*FvWRKY27*	*FvWRKY31*				0.7246	4.1163	0.1760
*FvWRKY27*	*FvWRKY52*				0.5880	2.9760	0.1976
*FvWRKY29*	*FvWRKY58* *				0.4700	2.2460	0.2093
*FvWRKY30*	*FvWRKY53*				0.4628	77.4216	0.0060
*FvWRKY39* *	*FvWRKY61* *				0.7849	2.7159	0.2890
*FvWRKY58* *	*FvWRKY64*				0.5863	63.4312	0.0092
**Tandem repeats**							
*FvWRKY11*	*FvWRKY12*				NA	NA	NA
*FvWRKY38*	*FvWRKY39* *	*FvWRKY40*	*FvWRKY41*	*FvWRKY42*	NA	NA	NA
*FvWRKY59*	*FvWRKY60*	*FvWRKY61* *			NA	NA	NA

**Table 2:** *FaWRKY* gene duplications. Nucleotide substitution values (Kn, Ks) and ω for tandemly duplicated genes were not provided by Synmap2. PAL2NAL was used to calculate Kn and Ks of the non-syntenic paralogs. ω values were not calculated if Ks < 0.01.

Gene 1	Gene 2	Gene 3	Kn	Ks	ω
**Segmental duplications**					
*FaWRKY6C.1*	*FaWRKY6C.2*		0.0026	0.0000	NA
*FaWRKY43B.1*	*FaWRKY43B.2*		0.0017	0.0055	NA
*FaWRKY54A.1*	*FaWRKY54A.2*		0.0000	0.0039	NA
*FaWRKY54D.1*	*FaWRKY54D.2*		0.0016	0.0000	NA
*FaWRKY56A.1*	*FaWRKY56A.2*		0.0083	0.0109	0.7615
*FaWRKY56D.1*	*FaWRKY56D.2*		0.0013	0.0047	NA
*FaWRKY57A.1*	*FaWRKY57A.2*		0.0000	0.0087	NA
*FaWRKY57D.1*	*FaWRKY57D.2*		0.0022	0.0000	NA
*FaWRKY58A.1*	*FaWRKY58A.2*		0.0064	0.0111	0.5766
*FaWRKY58D.1*	*FaWRKY58D.2*		0.0012	0.0043	NA
*FaWRKY63A.1*	*FaWRKY63A.2*		0.0024	0.0000	NA
*FaWRKY64A.1*	*FaWRKY64A.2*		0.0026	0.0063	NA
**Segmental, non-syntenic**					
*FaWRKY2D.1*	*FaWRKY2D.2*		0.0131	0.0153	0.8562
*FaWRKY2C.1*	*FaWRKY2C.2*		0.0000	0.0000	NA
*FaWRKY2C.1*	*FaWRKY2C.3*		0.0124	0.0147	0.8482
*FaWRKY2C.2*	*FaWRKY2C.3*		0.0124	0.0147	0.8482
*FaWRKY2B.1*	*FaWRKY2B.2*		0.0134	0.0153	0.8758
*FaWRKY2B.1*	*FaWRKY2B.3*		0.0125	0.0145	0.8621
*FaWRKY2B.2*	*FaWRKY2B.3*		0.0133	0.0103	1.2913
*FaWRKY14C.1*	*FaWRKY14C.2*		0.0071	0.0279	0.2545
*FaWRKY17C.1*	*FaWRKY17C.2*		0.0021	0.0084	NA
*FaWRKY18C.1*	*FaWRKY18C.2*		0.0072	0.0069	NA
*FaWRKY26B.1*	*FaWRKY26B.2*		0.3425	0.5631	0.6082
*FaWRKY29D.1*	*FaWRKY29D.2*		0.0011	0.0000	NA
*FaWRKY35D.1*	*FaWRKY35D.2*		0.0024	0.0101	0.2376
*FaWRKY36D.1*	*FaWRKY36D.2*		0.0190	0.0322	0.5901
*FaWRKY45D.1*	*FaWRKY45D.2*		0.0000	0.0000	NA
*FaWRKY47C.1*	*FaWRKY47C.2*		0.1398	0.2113	0.6616
*FaWRKY51A.1*	*FaWRKY51A.2*		0.0400	0.0457	0.8753
*FaWRKY51A.1*	*FaWRKY51A.3*		0.0965	0.1091	0.8845
*FaWRKY51A.2*	*FaWRKY51A.3*		0.0630	0.0586	1.0751
*FaWRKY62B.1*	*FaWRKY62B.2*		0.0298	0.0269	1.1078
*FaWRKY63D.1*	*FaWRKY63D.2*		0.0022	0.0053	NA
*FaWRKY63B.1*	*FaWRKY63B.2*		0.0147	0.0132	1.1136
*FaWRKY64D.1*	*FaWRKY64D.2*		0.0025	0.0032	NA
**Tandem duplications**					
*FaWRKY11C*	*FaWRKY12C*		NA	NA	NA
*FaWRKY11A*	*FaWRKY12A*		NA	NA	NA
*FaWRKY55B.1*	*FaWRKY55B.2*		NA	NA	NA
*FaWRKY38A*	*FaWRKY39A*	*FaWRKY41A*	NA	NA	NA
*FaWRKY38B*	*FaWRKY42B*		NA	NA	NA
*FaWRKY38C*	*FaWRKY42C*		NA	NA	NA
*FaWRKY40D*	*FaWRKY41D*	*FaWRKY42D*	NA	NA	NA

## Data Availability

All of the related sequence data in this study were downloaded from public databases and detailed in the Material and Methods. The datasets that support the conclusions of this article are included in this article.

## Supplementary Materials

The following supporting information can be downloaded at: www.mdpi.com/article/10.3390/plants11121585/s1, Figure S1: Original protein product of the snap_masked-Fvb3-3-processed-gene-269.16; Figure S2: Original and revised sequences for FaWRKY21C; Figure S3: Original and revised sequences for FaWRKY21B; Figure S4: Original and revised sequences for FaWRKY51A.2; Figure S5: Gene structure of strawberry WRKY family; Figure S6: Phylogenetic analysis of *Fragaria vesca* (Fv) and *Fragaria* × *ananassa* (Fa) WRKY proteins; Figure S7: Gene retention and fractionation bias in chromosomes 6 and 7 from *Fragaria vesca* and *Fragaria* × *ananassa* cv. Camarosa syntenic genes (1:4 syntenic depth); Figure S8: Expression profiles of *FvWRKY* family members in different tissues, developmental stages and growth conditions of *F. vesca*; Figure S9: Expression and hierarchical clustering of *FaWRKY* genes in strawberry achene and receptacle of green fruits (Green_A and Green_R, respectively), leaves and root tissues; Figure S10: Expression and hierarchical clustering of *FaWRKY* genes during strawberry fruit ripening in receptacle (A) and achene (B) tissues; Figure S11: Interaction network constructed with the differentially expressed FaWRKYs during ripening, based on experimental and co-expression data with high confidence (0.700) from their *Arabidopsis* orthologs; Figure S12: Expression and hierarchical clustering of *FaWRKY* genes in strawberry leaves, inoculated with mock or *C. fructicola* spores and collected at 24, 72 and 96 h; Table S1: FvWRKY annotations across all the available genome versions; Table S2: Main characteristics of the *Fragaria vesca WRKY* genes and proteins; Table S3: FaWRKY proteins characteristics; Table S4: Collinear relationships detected by SynMap3D among *F. vesca* (Fv), *A. thaliana* (At) and *V. vinifera* (Vv) *WRKY* genes; Table S5: Collinear relationships detected by SynMap2 between Fv-At and Fv-Vv *WRKY* genes; Table S6: Shared synteny between Fv and Fa WRKY orthologs; Table S7: Strawberry (Fv and Fa) WRKY classification into eggNOG hierarchical orthologous groups; Table S8: 23ggnog homologs to the *Arabidopsis* WRKY54/55/70 proteins; Table S9: *FvWRKY* expression values (TPM) in 46 strawberry tissues; Table S10: Putative *cis*-elements in the promoters of 257 *FaWRKY* genes; Table S11: Putative *cis*-elements in the promoters of 64 *FvWRKY* genes; Table S12: Raw counts of *FaWRKY* genes; Table S13: Expression values (log2 fold change, LFC) of differentially expressed *FaWRKY* genes in tissues, fruit ripening and defense response against *C. fructicola*; Table S14: RNA-seq expression z-scores computed for *FaWRKY* genes in tissues and hieralchical clusters; Table S15: RNA-seq expression z-scores computed for *FaWRKY* genes in fruit receptacles and hieralchical clusters; Table S16: RNA-seq expression z-scores computed for *FaWRKY* genes in fruit achenes and hieralchical clusters; Table S17: RNA-seq expression z-scores computed for *FaWRKY* genes in infected leaves and hieralchical clusters; Table S18: Lists of differentially expressed *FaWRKYs* in fruit tissues during any stage of ripening; Table S19: A selection of putative candidate *FaWRKY* genes for further studies; Table S20: Synteny analyses performed in CoGe and persistent links to results.

## References

[1] Eulgem T., Rushton P.J., Robatzek S., Somssich I.E. (2000). The WRKY superfamily of plant transcription factors. Trends Plant Sci..

[2] Bakshi M., Oelmuller R. (2014). WRKY transcription factors: Jack of many trades in plants. Plant Signal. Behav..

[3] Mohanta T.K., Park Y.H., Bae H. (2016). Novel Genomic and Evolutionary Insight of WRKY Transcription Factors in Plant Lineage. Sci. Rep..

[4] Zhang Y., Wang L. (2005). The WRKY transcription factor superfamily: Its origin in eukaryotes and expansion in plants. BMC Evol. Biol..

[5] Rushton P.J., Somssich I.E., Ringler P., Shen Q.J. (2010). WRKY transcription factors. Trends Plant Sci..

[6] Besseau S., Li J., Palva E.T. (2012). WRKY54 and WRKY70 co-operate as negative regulators of leaf senescence in *Arabidopsis thaliana*. J. Exp. Bot.

[7] Chen L., Xiang S., Chen Y., Li D., Yu D. (2017). *Arabidopsis* WRKY45 Interacts with the DELLA Protein RGL1 to Positively Regulate Age-Triggered Leaf Senescence. Mol. Plant.

[8] Doll J., Muth M., Riester L., Nebel S., Bresson J., Lee H.C., Zentgraf U. (2019). *Arabidopsis thaliana* WRKY25 Transcription Factor Mediates Oxidative Stress Tolerance and Regulates Senescence in a Redox-Dependent Manner. Front. Plant Sci..

[9] Zhang C.Q., Xu Y., Lu Y., Yu H.X., Gu M.H., Liu Q.Q. (2011). The WRKY transcription factor OsWRKY78 regulates stem elongation and seed development in rice. Planta.

[10] Zhang L., Chen L., Yu D. (2018). Transcription Factor WRKY75 Interacts with DELLA Proteins to Affect Flowering. Plant Physiol..

[11] Chen L., Song Y., Li S., Zhang L., Zou C., Yu D. (2012). The role of WRKY transcription factors in plant abiotic stresses. Biochim. Biophys. Acta.

[12] Tsuda K., Somssich I.E. (2015). Transcriptional networks in plant immunity. New Phytol..

[13] Alves M.S., Dadalto S.P., Goncalves A.B., de Souza G.B., Barros V.A., Fietto L.G. (2014). Transcription Factor Functional Protein-Protein Interactions in Plant Defense Responses. Proteomes.

[14] Ciolkowski I., Wanke D., Birkenbihl R.P., Somssich I.E. (2008). Studies on DNA-binding selectivity of WRKY transcription factors lend structural clues into WRKY-domain function. Plant Mol. Biol..

[15] Maeo K., Hayashi S., Kojima-Suzuki H., Morikami A., Nakamura K. (2001). Role of conserved residues of the WRKY domain in the DNA-binding of tobacco WRKY family proteins. Biosci. Biotechnol. Biochem..

[16] Cheng X., Zhao Y., Jiang Q., Yang J., Zhao W., Taylor I.A., Peng Y.L., Wang D., Liu J. (2019). Structural basis of dimerization and dual W-box DNA recognition by rice WRKY domain. Nucleic Acids Res..

[17] Singh A., Sharma A.K., Singh N.K., Sonah H., Deshmukh R., Sharma T.R. (2019). Understanding the Effect of Structural Diversity in WRKY Transcription Factors on DNA Binding Efficiency through Molecular Dynamics Simulation. Biology.

[18] Van Verk M.C., Pappaioannou D., Neeleman L., Bol J.F., Linthorst H.J. (2008). A Novel WRKY transcription factor is required for induction of PR-1a gene expression by salicylic acid and bacterial elicitors. Plant Physiol..

[19] Yang Y., Zhou Y., Chi Y., Fan B., Chen Z. (2017). Characterization of Soybean WRKY Gene Family and Identification of Soybean WRKY Genes that Promote Resistance to Soybean Cyst Nematode. Sci. Rep..

[20] Chen X., Li C., Wang H., Guo Z. (2019). WRKY transcription factors: Evolution, binding, and action. Phytopathol. Res..

[21] Machens F., Becker M., Umrath F., Hehl R. (2014). Identification of a novel type of WRKY transcription factor binding site in elicitor-responsive cis-sequences from *Arabidopsis thaliana*. Plant Mol. Biol..

[22] Rinerson C.I., Rabara R.C., Tripathi P., Shen Q.J., Rushton P.J. (2015). The evolution of WRKY transcription factors. BMC Plant Biol..

[23] DiMeglio L.M., Staudt G., Yu H., Davis T.M. (2014). A phylogenetic analysis of the genus *Fragaria* (strawberry) using intron-containing sequence from the ADH-1 gene. PLoS ONE.

[24] Shulaev V., Sargent D.J., Crowhurst R.N., Mockler T.C., Folkerts O., Delcher A.L., Jaiswal P., Mockaitis K., Liston A., Mane S.P. (2011). The genome of woodland strawberry (*Fragaria vesca*). Nat. Genet..

[25] Darwish O., Shahan R., Liu Z., Slovin J.P., Alkharouf N.W. (2015). Re-annotation of the woodland strawberry (*Fragaria vesca*) genome. BMC Genom..

[26] Tennessen J.A., Govindarajulu R., Ashman T.L., Liston A. (2014). Evolutionary origins and dynamics of octoploid strawberry subgenomes revealed by dense targeted capture linkage maps. Genome Biol. Evol..

[27] Li Y., Wei W., Feng J., Luo H., Pi M., Liu Z., Kang C. (2018). Genome re-annotation of the wild strawberry *Fragaria vesca* using extensive Illumina- and SMRT-based RNA-seq datasets. DNA Res..

[28] Edger P.P., VanBuren R., Colle M., Poorten T.J., Wai C.M., Niederhuth C.E., Alger E.I., Ou S., Acharya C.B., Wang J. (2018). Single-molecule sequencing and optical mapping yields an improved genome of woodland strawberry (*Fragaria vesca*) with chromosome-scale contiguity. Gigascience.

[29] Li Y., Pi M., Gao Q., Liu Z., Kang C. (2019). Updated annotation of the wild strawberry *Fragaria vesca* V4 genome. Hortic. Res..

[30] Edger P.P., Poorten T.J., VanBuren R., Hardigan M.A., Colle M., McKain M.R., Smith R.D., Teresi S.J., Nelson A.D.L., Wai C.M. (2019). Origin and evolution of the octoploid strawberry genome. Nat. Genet..

[31] Edger P.P., McKain M.R., Yocca A.E., Knapp S.J., Qiao Q., Zhang T. (2020). Reply to: Revisiting the origin of octoploid strawberry. Nat. Genet..

[32] Feng C., Wang J., Harris A.J., Folta K.M., Zhao M., Kang M. (2020). Tracing the diploid ancestry of the cultivated octoploid strawberry. Mol. Biol. Evol..

[33] Liston A., Wei N., Tennessen J.A., Li J., Dong M., Ashman T.L. (2020). Revisiting the origin of octoploid strawberry. Nat. Genet..

[34] Medina-Puche L., Blanco-Portales R., Molina-Hidalgo F.J., Cumplido-Laso G., Garcia-Caparros N., Moyano-Canete E., Caballero-Repullo J.L., Munoz-Blanco J., Rodriguez-Franco A. (2016). Extensive transcriptomic studies on the roles played by abscisic acid and auxins in the development and ripening of strawberry fruits. Funct. Integr. Genom..

[35] Sanchez-Sevilla J.F., Vallarino J.G., Osorio S., Bombarely A., Pose D., Merchante C., Botella M.A., Amaya I., Valpuesta V. (2017). Gene expression atlas of fruit ripening and transcriptome assembly from RNA-seq data in octoploid strawberry (*Fragaria* × *ananassa*). Sci. Rep..

[36] Amil-Ruiz F., Garrido-Gala J., Blanco-Portales R., Folta K.M., Munoz-Blanco J., Caballero J.L. (2013). Identification and validation of reference genes for transcript normalization in strawberry (*Fragaria* × *ananassa*) defense responses. PLoS ONE.

[37] Amil-Ruiz F., Garrido-Gala J., Gadea J., Blanco-Portales R., Munoz-Merida A., Trelles O., de Los Santos B., Arroyo F.T., Aguado-Puig A., Romero F. (2016). Partial Activation of SA- and JA-Defensive Pathways in Strawberry upon Colletotrichum acutatum Interaction. Front. Plant Sci..

[38] Wei W., Hu Y., Han Y.T., Zhang K., Zhao F.L., Feng J.Y. (2016). The WRKY transcription factors in the diploid woodland strawberry *Fragaria vesca*: Identification and expression analysis under biotic and abiotic stresses. Plant Physiol. Biochem..

[39] Zhou H., Li Y., Zhang Q., Ren S., Shen Y., Qin L., Xing Y. (2016). Genome-Wide Analysis of the Expression of WRKY Family Genes in Different Developmental Stages of Wild Strawberry (*Fragaria vesca*) Fruit. PLoS ONE.

[40] Solovyev V., Kosarev P., Seledsov I., Vorobyev D. (2006). Automatic annotation of eukaryotic genes, pseudogenes and promoters. Genome Biol..

[41] Panchy N., Lehti-Shiu M., Shiu S.H. (2016). Evolution of Gene Duplication in Plants. Plant Physiol..

[42] Lyons E., Freeling M. (2008). How to usefully compare homologous plant genes and chromosomes as DNA sequences. Plant J..

[43] Glover N.M., Redestig H., Dessimoz C. (2016). Homoeologs: What Are They and How Do We Infer Them?. Trends Plant Sci..

[44] Haas B.J., Delcher A.L., Wortman J.R., Salzberg S.L. (2004). DAGchainer: A tool for mining segmental genome duplications and synteny. Bioinformatics.

[45] Haug-Baltzell A., Stephens S.A., Davey S., Scheidegger C.E., Lyons E. (2017). SynMap2 and SynMap3D: Web-based whole-genome synteny browsers. Bioinformatics.

[46] Wang Q., Wang M., Zhang X., Hao B., Kaushik S.K., Pan Y. (2011). WRKY gene family evolution in *Arabidopsis thaliana*. Genetica.

[47] Chanderbali A.S., Berger B.A., Howarth D.G., Soltis D.E., Soltis P.S. (2017). Evolution of floral diversity: Genomics, genes and gamma. Philos. Trans. R. Soc. B Biol. Sci..

[48] Sahebi M., Hanafi M.M., van Wijnen A.J., Rice D., Rafii M.Y., Azizi P., Osman M., Taheri S., Bakar M.F.A., Isa M.N.M. (2018). Contribution of transposable elements in the plant’s genome. Gene.

[49] Bailey P.C., Schudoma C., Jackson W., Baggs E., Dagdas G., Haerty W., Moscou M., Krasileva K.V. (2018). Dominant integration locus drives continuous diversification of plant immune receptors with exogenous domain fusions. Genome Biol..

[50] Moore R.C., Purugganan M.D. (2005). The evolutionary dynamics of plant duplicate genes. Curr. Opin. Plant Biol..

[51] Vicient C.M., Casacuberta J.M. (2017). Impact of transposable elements on polyploid plant genomes. Ann. Bot..

[52] Bashir T., Chandra Mishra R., Hasan M.M., Mohanta T.K., Bae H. (2018). Effect of Hybridization on Somatic Mutations and Genomic Rearrangements in Plants. Int. J. Mol. Sci..

[53] Hughes A.L., Friedman R., Ekollu V., Rose J.R. (2003). Non-random association of transposable elements with duplicated genomic blocks in *Arabidopsis thaliana*. Mol. Phylogenet. Evol..

[54] Liu D., Hunt M., Tsai I.J. (2018). Inferring synteny between genome assemblies: A systematic evaluation. BMC Bioinform..

[55] Cooper G.M., Brown C.D. (2008). Qualifying the relationship between sequence conservation and molecular function. Genome Res..

[56] Jiao Y., Leebens-Mack J., Ayyampalayam S., Bowers J.E., McKain M.R., McNeal J., Rolf M., Ruzicka D.R., Wafula E., Wickett N.J. (2012). A genome triplication associated with early diversification of the core eudicots. Genome Biol..

[57] Murat F., Armero A., Pont C., Klopp C., Salse J. (2017). Reconstructing the genome of the most recent common ancestor of flowering plants. Nat. Genet..

[58] Xiang Y., Huang C.H., Hu Y., Wen J., Li S., Yi T., Chen H., Xiang J., Ma H. (2017). Evolution of Rosaceae Fruit Types Based on Nuclear Phylogeny in the Context of Geological Times and Genome Duplication. Mol. Biol. Evol..

[59] Koonin E.V. (2005). Orthologs, paralogs, and evolutionary genomics. Annu. Rev. Genet..

[60] Nehrt N.L., Clark W.T., Radivojac P., Hahn M.W. (2011). Testing the ortholog conjecture with comparative functional genomic data from mammals. PLoS Comput. Biol..

[61] APG (The Angiosperm Phylogeny Group) (2016). An update of the Angiosperm Phylogeny Group classification for the orders and families of flowering plants: APG IV. Bot. J. Linn. Soc..

[62] Tiley G.P., Ane C., Burleigh J.G. (2016). Evaluating and Characterizing Ancient Whole-Genome Duplications in Plants with Gene Count Data. Genome Biol. Evol..

[63] Kim K.D., El Baidouri M., Abernathy B., Iwata-Otsubo A., Chavarro C., Gonzales M., Libault M., Grimwood J., Jackson S.A. (2015). A Comparative Epigenomic Analysis of Polyploidy-Derived Genes in Soybean and Common Bean. Plant Physiol..

[64] Schmutz J., Cannon S.B., Schlueter J., Ma J., Mitros T., Nelson W., Hyten D.L., Song Q., Thelen J.J., Cheng J. (2010). Genome sequence of the palaeopolyploid soybean. Nature.

[65] Velasco R., Zharkikh A., Affourtit J., Dhingra A., Cestaro A., Kalyanaraman A., Fontana P., Bhatnagar S.K., Troggio M., Pruss D. (2010). The genome of the domesticated apple (*Malus* x *domestica* Borkh.). Nat. Genet..

[66] Luo M.C., You F.M., Li P., Wang J.R., Zhu T., Dandekar A.M., Leslie C.A., Aradhya M., McGuire P.E., Dvorak J. (2015). Synteny analysis in Rosids with a walnut physical map reveals slow genome evolution in long-lived woody perennials. BMC Genom..

[67] Chen J., Nolan T.M., Ye H., Zhang M., Tong H., Xin P., Chu J., Chu C., Li Z., Yin Y. (2017). Arabidopsis WRKY46, WRKY54, and WRKY70 Transcription Factors Are Involved in Brassinosteroid-Regulated Plant Growth and Drought Responses. Plant Cell.

[68] Li J., Zhong R., Palva E.T. (2017). WRKY70 and its homolog WRKY54 negatively modulate the cell wall-associated defenses to necrotrophic pathogens in Arabidopsis. PLoS ONE.

[69] Amborella Genome P. (2013). The Amborella genome and the evolution of flowering plants. Science.

[70] Lei Y., Sun Y., Wang B., Yu S., Dai H., Li H., Zhang Z., Zhang J. (2020). Woodland strawberry WRKY71 acts as a promoter of flowering via a transcriptional regulatory cascade. Hortic. Res..

[71] Wei W., Cui M.Y., Hu Y., Gao K., Xie Y.G., Jiang Y., Feng J.Y. (2018). Ectopic expression of FvWRKY42, a WRKY transcription factor from the diploid woodland strawberry (*Fragaria vesca*), enhances resistance to powdery mildew, improves osmotic stress resistance, and increases abscisic acid sensitivity in *Arabidopsis*. Plant Sci. An. Int. J. Exp. Plant Biol..

[72] Zhang W.W., Zhao S.Q., Gu S., Cao X.Y., Zhang Y., Niu J.F., Liu L., Li A.R., Jia W.S., Qi B.X. (2022). FvWRKY48 binds to the pectate lyase FvPLA promoter to control fruit softening in *Fragaria vesca*. Plant Physiol..

[73] Hawkins C., Caruana J., Li J., Zawora C., Darwish O., Wu J., Alkharouf N., Liu Z. (2017). An eFP browser for visualizing strawberry fruit and flower transcriptomes. Hortic. Res..

[74] Hollender C.A., Geretz A.C., Slovin J.P., Liu Z. (2012). Flower and early fruit development in a diploid strawberry, *Fragaria vesca*. Planta.

[75] Toljamo A., Blande D., Karenlampi S., Kokko H. (2016). Reprogramming of Strawberry (*Fragaria vesca*) Root Transcriptome in Response to *Phytophthora cactorum*. PLoS ONE.

[76] Hussain R.M.F., Sheikh A.H., Haider I., Quareshy M., Linthorst H.J.M. (2018). Arabidopsis WRKY50 and TGA Transcription Factors Synergistically Activate Expression of PR1. Front. Plant Sci..

[77] Gao Q.M., Venugopal S., Navarre D., Kachroo A. (2011). Low oleic acid-derived repression of jasmonic acid-inducible defense responses requires the WRKY50 and WRKY51 proteins. Plant Physiol..

[78] Chen L., Zhang L., Xiang S., Chen Y., Zhang H., Yu D. (2021). The transcription factor WRKY75 positively regulates jasmonate-mediated plant defense to necrotrophic fungal pathogens. J. Exp. Bot..

[79] Encinas-Villarejo S., Maldonado A.M., Amil-Ruiz F., de los Santos B., Romero F., Pliego-Alfaro F., Muñoz-Blanco J., Caballero J.L. (2009). Evidence for a positive regulatory role of strawberry (*Fragaria* × *ananassa*) Fa WRKY1 and *Arabidopsis* At WRKY75 proteins in resistance. J. Exp. Bot..

[80] Higuera J.J., Garrido-Gala J., Lekhbou A., Arjona-Girona I., Amil-Ruiz F., Mercado J.A., Pliego-Alfaro F., Munoz-Blanco J., Lopez-Herrera C.J., Caballero J.L. (2019). The Strawberry FaWRKY1 Transcription Factor Negatively Regulates Resistance to Colletotrichum acutatum in Fruit Upon Infection. Front. Plant Sci..

[81] Eulgem T. (2006). Dissecting the WRKY web of plant defense regulators. PLoS Pathog..

[82] Wang D., Amornsiripanitch N., Dong X. (2006). A genomic approach to identify regulatory nodes in the transcriptional network of systemic acquired resistance in plants. PLoS Pathog..

[83] Birkenbihl R.P., Kracher B., Ross A., Kramer K., Finkemeier I., Somssich I.E. (2018). Principles and characteristics of the *Arabidopsis* WRKY regulatory network during early MAMP-triggered immunity. Plant J..

[84] Zheng Z., Mosher S.L., Fan B., Klessig D.F., Chen Z. (2007). Functional analysis of *Arabidopsis* WRKY25 transcription factor in plant defense against *Pseudomonas syringae*. BMC Plant Biol..

[85] Roulin A., Auer P.L., Libault M., Schlueter J., Farmer A., May G., Stacey G., Doerge R.W., Jackson S.A. (2013). The fate of duplicated genes in a polyploid plant genome. Plant J..

[86] Zhang L., Huang X., He C., Zhang Q.Y., Zou X., Duan K., Gao Q. (2018). Novel Fungal Pathogenicity and Leaf Defense Strategies Are Revealed by Simultaneous Transcriptome Analysis of *Colletotrichum fructicola* and Strawberry Infected by This Fungus. Front. Plant Sci..

[87] Devaiah B.N., Karthikeyan A.S., Raghothama K.G. (2007). WRKY75 transcription factor is a modulator of phosphate acquisition and root development in *Arabidopsis*. Plant Physiol..

[88] Garrido-Gala J., Higuera J.J., Munoz-Blanco J., Amil-Ruiz F., Caballero J.L. (2019). The VQ motif-containing proteins in the diploid and octoploid strawberry. Sci. Rep..

[89] Medina-Puche L., Martinez-Rivas F.J., Molina-Hidalgo F.J., Mercado J.A., Moyano E., Rodriguez-Franco A., Caballero J.L., Munoz-Blanco J., Blanco-Portales R. (2019). An atypical HLH transcriptional regulator plays a novel and important role in strawberry ripened receptacle. BMC Plant Biol..

[90] Zhao F., Li G., Hu P., Zhao X., Li L., Wei W., Feng J., Zhou H. (2018). Identification of basic/helix-loop-helix transcription factors reveals candidate genes involved in anthocyanin biosynthesis from the strawberry white-flesh mutant. Sci. Rep..

[91] Bhattarai K.K., Atamian H.S., Kaloshian I., Eulgem T. (2010). WRKY72-type transcription factors contribute to basal immunity in tomato and *Arabidopsis* as well as gene-for-gene resistance mediated by the tomato R gene Mi-1. Plant J..

[92] Van Verk M.C., Bol J.F., Linthorst H.J. (2011). WRKY transcription factors involved in activation of SA biosynthesis genes. BMC Plant Biol..

[93] Hu Y., Dong Q., Yu D. (2012). *Arabidopsis* WRKY46 coordinates with WRKY70 and WRKY53 in basal resistance against pathogen *Pseudomonas syringae*. Plant Sci. Int. J. Exp. Plant Biol..

[94] Li J., Brader G., Kariola T., Palva E.T. (2006). WRKY70 modulates the selection of signaling pathways in plant defense. Plant J..

[95] Li J., Brader G., Palva E.T. (2004). The WRKY70 transcription factor: A node of convergence for jasmonate-mediated and salicylate-mediated signals in plant defense. Plant Cell.

[96] Birkenbihl R.P., Kracher B., Roccaro M., Somssich I.E. (2017). Induced Genome-Wide Binding of Three *Arabidopsis* WRKY Transcription Factors during Early MAMP-Triggered Immunity. Plant Cell.

[97] Pandey S.P., Roccaro M., Schon M., Logemann E., Somssich I.E. (2010). Transcriptional reprogramming regulated by WRKY18 and WRKY40 facilitates powdery mildew infection of *Arabidopsis*. Plant J..

[98] Zheng Z., Qamar S.A., Chen Z., Mengiste T. (2006). *Arabidopsis* WRKY33 transcription factor is required for resistance to necrotrophic fungal pathogens. Plant J..

[99] Wang F., Zhang F., Chen M., Liu Z., Zhang Z., Fu J., Ma Y. (2017). Comparative Transcriptomics Reveals Differential Gene Expression Related to Colletotrichum gloeosporioides Resistance in the Octoploid Strawberry. Front. Plant Sci..

[100] Mukhi N., Brown H., Gorenkin D., Ding P., Bentham A.R., Stevenson C.E.M., Jones J.D.G., Banfield M.J. (2021). Perception of structurally distinct effectors by the integrated WRKY domain of a plant immune receptor. Proc. Natl. Acad. Sci. USA.

[101] Narusaka M., Shirasu K., Noutoshi Y., Kubo Y., Shiraishi T., Iwabuchi M., Narusaka Y. (2009). RRS1 and RPS4 provide a dual Resistance-gene system against fungal and bacterial pathogens. Plant J..

[102] Jung S., Lee T., Cheng C.H., Buble K., Zheng P., Yu J., Humann J., Ficklin S.P., Gasic K., Scott K. (2019). 15 years of GDR: New data and functionality in the Genome Database for Rosaceae. Nucleic Acids Res..

[103] El-Gebali S., Mistry J., Bateman A., Eddy S.R., Luciani A., Potter S.C., Qureshi M., Richardson L.J., Salazar G.A., Smart A. (2019). The Pfam protein families database in 2019. Nucleic Acids Res..

[104] Okonechnikov K., Golosova O., Fursov M., Ugene Team (2012). Unipro UGENE: A unified bioinformatics toolkit. Bioinformatics.

[105] Marchler-Bauer A., Bo Y., Han L., He J., Lanczycki C.J., Lu S., Chitsaz F., Derbyshire M.K., Geer R.C., Gonzales N.R. (2017). CDD/SPARCLE: Functional classification of proteins via subfamily domain architectures. Nucleic Acids Res..

[106] Guo C., Guo R., Xu X., Gao M., Li X., Song J., Zheng Y., Wang X. (2014). Evolution and expression analysis of the grape (*Vitis vinifera* L.) WRKY gene family. J. Exp. Bot..

[107] Jin J., Tian F., Yang D.C., Meng Y.Q., Kong L., Luo J., Gao G. (2017). PlantTFDB 4.0: Toward a central hub for transcription factors and regulatory interactions in plants. Nucleic Acids Res..

[108] Voorrips R.E. (2002). MapChart: Software for the graphical presentation of linkage maps and QTLs. J. Hered..

[109] Sperschneider J., Catanzariti A.M., DeBoer K., Petre B., Gardiner D.M., Singh K.B., Dodds P.N., Taylor J.M. (2017). LOCALIZER: Subcellular localization prediction of both plant and effector proteins in the plant cell. Sci. Rep..

[110] Afgan E., Baker D., van den Beek M., Blankenberg D., Bouvier D., Cech M., Chilton J., Clements D., Coraor N., Eberhard C. (2016). The Galaxy platform for accessible, reproducible and collaborative biomedical analyses: 2016 update. Nucleic Acids Res..

[111] Kumar S., Stecher G., Tamura K. (2016). MEGA7: Molecular Evolutionary Genetics Analysis Version 7.0 for Bigger Datasets. Mol. Biol. Evol..

[112] Letunic I., Bork P. (2019). Interactive Tree Of Life (iTOL) v4: Recent updates and new developments. Nucleic Acids Res..

[113] Suyama M., Torrents D., Bork P. (2006). PAL2NAL: Robust conversion of protein sequence alignments into the corresponding codon alignments. Nucleic Acids Res..

[114] Yang Z., Nielsen R. (2002). Codon-substitution models for detecting molecular adaptation at individual sites along specific lineages. Mol. Biol. Evol..

[115] Villanueva-Canas J.L., Laurie S., Alba M.M. (2013). Improving genome-wide scans of positive selection by using protein isoforms of similar length. Genome Biol. Evol..

[116] De La Torre A.R., Li Z., Van de Peer Y., Ingvarsson P.K. (2017). Contrasting Rates of Molecular Evolution and Patterns of Selection among Gymnosperms and Flowering Plants. Mol. Biol. Evol..

[117] Huerta-Cepas J., Forslund K., Coelho L.P., Szklarczyk D., Jensen L.J., von Mering C., Bork P. (2017). Fast Genome-Wide Functional Annotation through Orthology Assignment by eggNOG-Mapper. Mol. Biol. Evol..

[118] Huerta-Cepas J., Szklarczyk D., Heller D., Hernandez-Plaza A., Forslund S.K., Cook H., Mende D.R., Letunic I., Rattei T., Jensen L.J. (2019). eggNOG 5.0: A hierarchical, functionally and phylogenetically annotated orthology resource based on 5090 organisms and 2502 viruses. Nucleic Acids Res..

[119] Ye J., Zhang Y., Cui H., Liu J., Wu Y., Cheng Y., Xu H., Huang X., Li S., Zhou A. (2018). WEGO 2.0: A web tool for analyzing and plotting GO annotations, 2018 update. Nucleic Acids Res..

[120] Szklarczyk D., Gable A.L., Nastou K.C., Lyon D., Kirsch R., Pyysalo S., Doncheva N.T., Legeay M., Fang T., Bork P. (2020). The STRING database in 2021: Customizable protein–protein networks, and functional characterization of user-uploaded gene/measurement sets. Nucleic Acids Res..

[121] Kopylova E., Noe L., Touzet H. (2012). SortMeRNA: Fast and accurate filtering of ribosomal RNAs in metatranscriptomic data. Bioinformatics.

[122] Dobin A., Davis C.A., Schlesinger F., Drenkow J., Zaleski C., Jha S., Batut P., Chaisson M., Gingeras T.R. (2013). STAR: Ultrafast universal RNA-seq aligner. Bioinformatics.

