# Supplementary material for: A Comprehensive Study of the WRKY Transcription Factor Family in Strawberry
Source: Plants (Basel). 2022 Jun 15;11(12):1585. doi: 10.3390/plants11121585 (PMC9229891; doi:10.3390/plants11121585)
Supplement: Supplementary file 1 [file plants-11-01585-s001.zip › Suppl figures.pdf]

>snap_masked-Fvb3-3-processed-gene-269.16 protein (original, truncated)
MAGMDDNVAIIGDWVPPSPSPRDFFSSMFVDGIGSRSFLDSPNSNKAEDFFLGSREGSTSGKN
LLQGNGTGDEITEMGSFSEYKSNTRGGLVERIAARAGFNAPRLNTESIRSSDLSLSSDVRSPYLTIP
PGLSPTTLLDSPVFLLNSLAQPSPTTGKFPFVSNGHSRNSTLMTEAADNANFFEDLNSSFAFKPL
GESGSFFLGPTSKTT

>FaWRKY20A protein (revised)
MAGMDDNVAIIGDWVPPSPSPRDFFSSMFVDGIGSRSFLDSPNSNKAEDFFLGSREGSTSGKN
LLQGNGTGDEITEMGSFSEYKSNTRGGLVERIAARAGFNAPRLNTESIRSSDLSLSSDVRSPYLTIP
PGLSPTTLLDSPVFLLNSLAQPSPTTGKFPFVSNGHSRNSTLMTEAADNANFFEDLNSSFAFKPL
GESGSFFLVGGSTEHSPPPDEQPEEEGDQRGGGDSMAATGGGTPSEDGYN**WRKYGQKQVKG**
SEYPRSYYKCTHPNCQVKKKVERSHEGHITEIIYKGAHNHSKPPPNRRSGAIGSSNPLIDMRSD
VLEQGGPQTGADGDLVWASAQKATGGAPDWKHDNLEVTSSASAGPDYGQQSTSMQAQNG
AHLESGDVVDASSTFSNDEDEDDRGTHGSVSLVYDGEGDESESKRRKIEAYATEMSGATRAIREP
RVVVQTTSEVDILDDGYR**WRKYGQKVVKGNPNPRSYYKCTNAGCTVRKHVERASHDLKSVIT**
TYEGKHNHDVPAARNSSHVNSGPSGNMSGQVSGSATQTHPHRPEPSQVHNSMARFERPMS
MGSFSLPGRQQLGPPQGFSFGMNQPGLANLAMAGFGPGQHKVPVLPVHHPYFSQQRQVSE
MGFMLPKGEPKVEPMSEPGLNMSNASSVYQQLMSRLPLGPQM

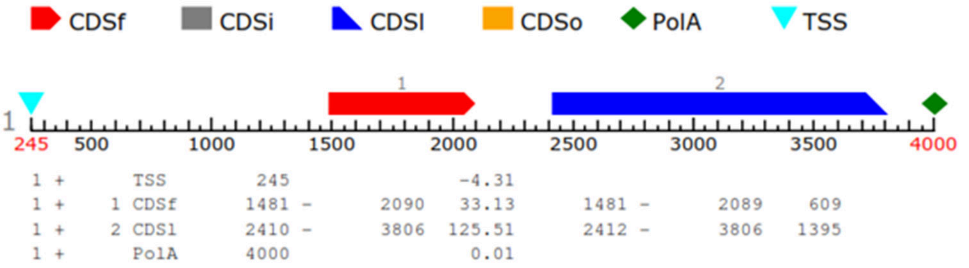

Figure S1. Original protein product of the snap_masked-Fvb3-3-processed-gene-269.16. This gene was identified as syntenic with other *FaWRKY* genes, but the originally predicted protein did not harbor WRKY domains. The source mRNA was loaded in FGENESH and a revised protein sequence was generated.

>maker-Fvb3-1-snap-gene-3.50-mRNA-1 (original)
CTTCGTCCTAGTTAGCTGGCTGGCTAATTTATACATTATTTCCCAACTCAGGGTTATATATCAGAAAG
AGTGATACTTGCTTTGCTGCTCCGATCTATCTTTGCCCTCTTGAGTCTTAAGCTTACATACAAATTAC
ACAGCGAGGAAGGGAGGGTTGTATTTACAGTGTTTCTGGATAGAATCGCAGCCAAGAACAGTCG
TAGGACTAAAGTGGTGAACCATATTTTGATTCTCATCAGTCTCTCAGAAAATATACACATAAAAGGG
AAAACTGATCATCATGATGAGGATGAGGGACATGGAGAAGGAGAAGAGGAGGTGCAAGACAAT
ACCAACTCAGGCGGAAGCAACAAAGTGGTGATACCAGAAGATGGCTTCGAGTGGAGAAAATATG
GACAGAAGTTTATTAAAAATATTGGAAAGTTCAGAAGCTATTTCAAGTGCCAAAACAGCAATTGTA
GAGCCAAGAAGCGAGCCGAGTGGTCGAGCTCAGAGCCTGATAAACTTCGAGTAGTGTATGAAGG
GGTGCACAATCACACTGATAATAATGCCTCATCTGCAGGATCATCGACTTCACATTCCTCATCATCTT
TAAATCCGAGTGCTGATGCAAACCAATATGACTTGTACACACAAGTTTTTGGTAACCAATCCTCCCA
TTGGGATAAAAAAAGTTGAATCTGATAGCTGCAGCAGCAGGTTAATCCACACAGTGCATGACTACC
AGTTCTTCTGCAGCTGTTATTTACATCCCTGTTGGATAAGCCTCGCCATTTCAATATATAAGTTTAATG
CCAGTCTGTAATTGCGGTGTAAAGAAACAAGTATATCATTTGGTCCATCTATATCCGCTAATAATTTC
ATTACTGGTACAACTGATCCTGAACTCTTGGAAACAATGTCCAAGGTCAATTGACCAGCAGATATAC
AGTGTCGTATCCATATGCAAAGTTGAATAGGGTGACATACAGCATATACGTTATTGTTGAGATTGCG
CTTGCAATGCTATTGGTC
>maker-Fvb3-1-snap-gene-3.50-mRNA-1 protein (original)
MQTNMTCTHKFLVTNPPIGIKKVESDSCSSRLIHTVHDYQFFCSCYLHPCWISLAISIYKFNASL

>FaWRKY21C cDNA (revised)
ATGTCCGATAATGATTTGATTTTATATAGGAAAACTGATCATCATGATGAGGATGAGGGACATGGAG
AAGGAGAAGAGGAGGTGCAAGACAATACCAACTCAGGCGGAAGCAACAAAGTGGTGATACCAG
AAGATGGCTTCGAGTGGAGAAAATATGGACAGAAGTTTATTAAAAATATTGGAAAGTTCAGAAGC
TATTTCAAGTGCCAAAACAGCAATTGTAGAGCCAAGAAGCGAGCCGAGTGGTCGAGCTCAGAGC
CTGATAAACTTCGAGTAGTGTATGAAGGGGTGCACAATCACACTGATAATAATGCCTCATCTGCAG
GATCATCGACTTCACATTCCTCATCATCTTTAAATCCGAGTGCTGATGCAAACCAATATGACTTGTAC
ACACAAGTTTTTGGTAACCAATCCTCCCATTGGGATAAAAAAAGTTGA
>FaWRKY21C protein (revised)
MSDNDLILYRKTDHHDEDEGHGEGEEEVQDNTNSGGSNKVVIPEDGFE**WRKYGQKFIKNIGKFRSY
FKCQNSNCRAKKRAEWSSSEPDKLRVVYEGVHNH**TDNNASSAGSSTSHSSSSLNPSADANQYDLYT
QVFGNQSSHWDKKS

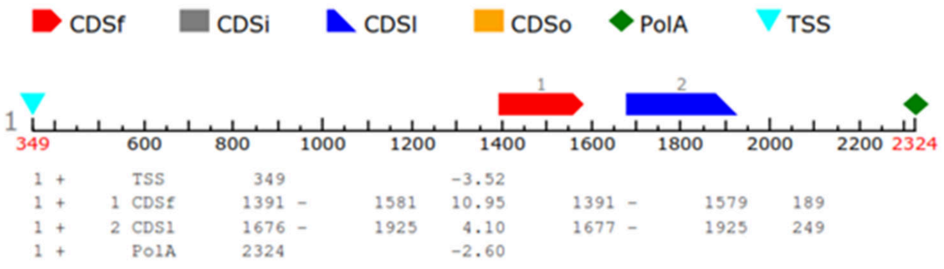

Figure S2. Original and revised sequences for FaWRKY21C.

>maker-Fvb3-2-snap-gene-310.32-mRNA-1 (original)
TATCAAGTAGCGAGTGGACACGTTCAGGTTTCATTGGCTGAGAATGTGATTCCGAGCCGTATAGCAG
CTGGTGCTGAGGTTATATATCAGAAAGAGCATAGAGTGATACTTGCTTTGCTGCTCCGATCTATCTCT
GCCCTCTTGAGTCTTAAGCTTACATACAAATTACACAGCGAGGAAGGGAGGGTTGTATTTACAGTGT
TTCTGGATAGAATCGCAGCCACGAGCAGTCGTAGGACTAAAGTGGTGAACCATGATGAGGGAGATG
GAGAAGGAGAAGAGGAGGTGCAAGACAACAACAATTCAGGCGGAAGCAACAAAGTGGTGATACC
AGAAGATGGCTTCGAGTGGAAAAAATATGGACAGAAGTTTATTAAAAATGTTGGAAAGTTCAGAAG
CTATTTCAAGTGCCAAAACAGCAATTGTAGAGCCAAGAAGCGAGCAGAGTGGTCGAGCTCAAAGC
CTGATAAACTTCGAGTAGTGTATGAAGGGGTGCACAATCACACTGATAATAATGCCTCATCTGCAGG
ATCATCGACATCACATTCCTCATCATCTTTAAATCCGAGTGCTGATGCAAACCAATATGACTTGTACAC
ACAAGTTTTTGGTAACCAATCCTCCCATTGGGATAAAAAAAGTTGAATCTTCTAGCTGCAGCAGCAG
GTTAATCCACACAGTGCATGACTACCAGTTCTTCTGCAGCTGTTATTTACATCCCTCTTAGATAAGCCT
CGCCATTTCAATATATAAGTTTAATGCCAGTCTGTAATTGCGGAGCAAAGAAACAAGTATATCATTTG
GTCCATCTATATCCGCTAATAATTTCATTACTGGTACAACTGATCCTGAACTCTTGGAGACAATGTCCA
AGGTCAATTAACCAGCAGATATACAAAGTGTCTTGTCCATACGCAAAGTTGAATAGGG
>maker-Fvb3-2-snap-gene-310.32 protein (original)
YQVASGHVQVSLAENVIPSRIAAGAEVIYQKEHRVILALLLRSISALLSLKLTYKLHSEEGRVVFTVFLDRIA
ATSSRRTKVVNHDEGDGEGEEEVQDNNNSGGSNKVVIPEDGFE**WKKYGQKFIKNVGKFRSYFKCQN**
SNCRAKKRAEWSSSKPDKLRVVYEGVHNHTDNNASSAGSSTSHSSSSLNPSADANQYDLYTQVFGN
QSSHWDKKS

>FaWRKY21B cDNA (revised)
ATGTCCGATTATGATTTGATTTTATATAGGAAAACTGATCATCATGATGAGGATGAGGGAGATGGAGA
AGGAGAAGAGGAGGTGCAAGACAACAACAATTCAGGCGGAAGCAACAAAGTGGTGATACCAGAA
GATGGCTTCGAGTGGAAAAAATATGGACAGAAGTTTATTAAAAATGTTGGAAAGTTCAGAAGCTAT
TTCAAGTGCCAAAACAGCAATTGTAGAGCCAAGAAGCGAGCAGAGTGGTCGAGCTCAAAGCCTGA
TAAACTTCGAGTAGTGTATGAAGGGGTGCACAATCACACTGATAATAATGCCTCATCTGCAGGATCAT
CGACATCACATTCCTCATCATCTTTAAATCCGAGTGCTGATGCAAACCAATATGACTTGTACACACAA
GTTTTTGGTAACCAATCCTCCCATTGGGATAAAAAAAGTTGA
> FaWRKY21B protein (revised)
MSDYDLILYRKTDHHDEDEGDGEGEEEVQDNNNSGGSNKVVIPEDGFE**WKKYGQKFIKNVGKFRSYF**
KCQNSNCRAKKRAEWSSSKPDKLRVVYEGVHNHTDNNASSAGSSTSHSSSSLNPSADANQYDLYTQ
VFGNQSSHWDKKS

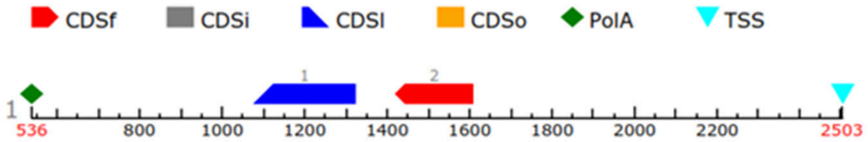

1 -		PolA	536		2.20			
1 -	1	CDSl	1073 -	1322	5.23	1073 -	1321	249
1 -	2	CDSf	1417 -	1607	12.84	1419 -	1607	189
1 -		TSS	2503		-3.61			

Figure S3. Original and revised sequences for FaWRKY21B

>maker-Fvb6-2-snap-gene-308.69-mRNA-1 (original)
TATATTGTCCATTTTTCATTTCTGCTGCGAGGACTTTCATCCCCACTTGAAATTCAGGATAGCACGAAACCCACAGAGAACA
AAACCACCCAACCGAGCAGTTGCAGAGGAAGAAAGAAAGAAGAAACAAGCATATACTTTGAAGAAGATAACATAACAG
AGTACTTCTTCTACAAAAACAGAGGATTTCAGAAACAGAGCACATCGGTAAAGCTCCCAACTTTAATGGCGGCTAAGCAC
CAAGACTCGATGTCAGCACCGCAGCCTCAGCGGCCGGTGATATCACTCCCTCCGAGGCCTTCCGCGGAGTCCCTTTTCGG
CGGCGGATCCGGAACCAGCCCAGGTCCGATGACTCTGGTCTCAAGCTTCTTCTCAGACTCAGGATTTCAGGTCCTTCTCT
CAGCTTCTTGCTGGAGCTATGGGTCACCTTTGGCTCAAGTTAGGCCTATTATGTTTGGGCAGAACCAAAACCCAGTTAGG
AATGAAGGTGCTGGGTCTGAAAATGGAGGGGAGAAGAGTTCTGGGTTTAAGCAGAGTAGGCCTATGAATTTGATGGTG
ACTCACTCGCCATTGTTTACTGTCCCACCTGGGTTGAGCCCCTCCGGGTTGCTTAACTCGCCTGCCGGGTTCTTTGCACCT
CCGAGCCCATTTGGAATATCACACCAGCAGGCCTTGGCACATGTCACTGCACTAGCTCAATCTAATTTGCAGATGCAAGCT
GAATATCAATCTTCATTACTAGCAGCTCCCATAGAGTCGCAGCCAAGTAATGCTTCCTCAATCCCCAATGAAGCTTCTCAAC
AGCAAACAACACCCTCAACATCTGAACTTGGAAGTTCCATGGTACAAACATCAGAGGCTTCTCATTCTGATAGAAAATACC
CTTCATCCCATGCCACCGATAAACCTGCAGATGATCGTTATAACTGGCGAAAATATGGGCAGAAGCAGGTTAAGGGGAGT
GAATATCCACGAAGCTACTATAAATGTACACATGTGAACTGCCCTGTCAAAAGAAAGTTGAGCGTTCACCTGCCGGCGAA
ATAACTGAGATTATCTACAAAGGGCAACATAACCATGAGCCACCTCAACCTAATAAACGTGTGAAAGATGGAGGTGGTCA
GAATGGACATATGGATTCACAGCCTAAGCTTGAAAATGGTTTACAAAGAAGAGTCGGAGATTCAACAAATCATGTGAAGC
TGTGCCTGAGAGGGATTATGAGCATACTCAAGCTGCTCCTGTGCAGTTACCAAGTGACAGTGAAGACTTGGGTGATGCG
GAAGCCAGAGAAGAAGGGGATGTTGATGAACCTAATCCAAAGAGAAGGAACATTGATGGTGTCTCATCTGAGGTAGCTT
TGCCTCACAAGACGGTGACAGAGCCTAAAATCATAGTTCAACAAGGAGTGAAGTTGATCTCCTTGATGATGGTTACAGAT
GGAGAAAGTATGGGCAGAAAGTAGTCAAAGGGAATCCTCATCCAAG
>maker-Fvb6-2-snap-gene-308.69 protein (original)
YIVHFSFLLRGLSSPLEIQDSTKPTENKTTQPSSCRGRKKEETSIYFEEDNITEYFFYKNRGFQKQSTSVKLPTLMAAKHQDSMSA
PQPQRPVISLPPRPSAESLFGGGSGTSPGPMTLVSSFFSDSGFQVLLSASCWSYGSPLAQVRPIMFGQNQNPVRNEGAGSEN
GGEKSSGFKQSRPMNLMVTHSPLFTVPPGLSPSGLLNSPAGFFAPPSPFGISHQQALAHVTALAQSNLQMQAEYQSSLLAAPI
ESQPSNASSIPNEASQQQTTPSTSELGSSMVQTSEASHSDRKYPSSHATDKPADDRYN**WRKYGQK**QVKGSEYPRSYYKCTHV
NCPVKRKLSVHLPAK

>FaWRKY51A.2 cDNA (revised)
ATGGCGGCTAAGCACCAAGACTCGATGTCAGCACCGCAGCCTCAGCGGCCGGTGATATCACTCCCTCCGAGGCCTTCCGC
GGAGTCCCTTTTCGGCGGCGGATCCGGAACCAGCCCAGCTTCTTGCTGGAGCTATGGGTCACCTTTGGCTCAAGTTAGG
CCTATTATGTTTGGGCAGAACCAAAACCCAGTTAGGAATGAAGGTGCTGGGTCTGAAAATGGAGGGGAGAAGAGTTCT
GGGTTTAAGCAGAGTAGGCCTATGAATTTGATGGTGACTCACTCGCCATTGTTTACTGTCCCACCTGGGTTGAGCCCCTCC
GGGTTGCTTAACTCGCCTGCCGGGTTCTTTGCACCTCCGAGCCCATTTGGAATATCACACCAGCAGGCCTTGGCACATGT
CACTGCACTAGCTCAATCTAATTTGCAGATGCAAGCTGAATATCAATCTTCATTACTAGCAGCTCCCATAGAGTCGCAGCCA
AGTAATGCTTCCTCAATCCCCAATGAAGCTTCTCAACAGCAAACAACACCCTCAACATCTGAACTTGGAAGTTCCATGGTA
CAAACATCAGAGGCTTCTCATTCTGATAGAAAATACCCTTCATCCCATGCCACCGATAAACCTGCAGATGATCGTTATAACT
GGCGAAAATATGGGCAGAAGCAGAAGAGTCGGAGATTCAACAAATCATGTGAAGCTGTGCCTGAGAGGGATTATGAGC
ATACTCAAGCTGCTCCTGTGCAGTTACCAAGTGACAGTGAAGACTTGGGTGATGCGGAAGCCAGAGAAGAAGGGGATG
TTGATGAACCTAATCCAAAGAGAAGGAACATTGATGGTGTCTCATCTGAGGTAGCTTTGCCTCACAAGACGGTGACAGAG
CCTAAAATCATAGTTCAACAAGGAGTGAAGTTGATCTCCTTGATGATGGTTACAGATGGAGAAAGTATGGGCAGAAAGTA
G
>FaWRKY51A.2 protein (revised)
MAAKHQDSMSAPQPQRPVISLPPRPSAESLFGGGSGTSPASCWSYGSPLAQVRPIMFGQNQNPVRNEGAGSENGGEKSSG
FKQSRPMNLMVTHSPLFTVPPGLSPSGLLNSPAGFFAPPSPFGISHQQALAHVTALAQSNLQMQAEYQSSLLAAPIESQPSNA
SSIPNEASQQQTTPSTSELGSSMVQTSEASHSDRKYPSSHATDKPADDRYN**WRKYGQK**QKSRRFNKSCEAVPERDYEHTQA
APVQLPSDSEDLGDAEAREEGDVDEPNPKRRNIDGVSSEVALPHKTVTEPKIIVQQGVKLISLMMVTDGESMGRK

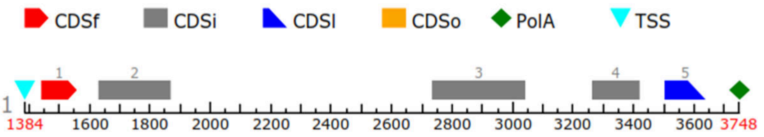

1	+		TSS	1384			-0.03				
1	+	1	CDSf	1439	-	1556	20.62	1439	-	1555	117
1	+	2	CDSi	1628	-	1866	23.15	1630	-	1866	237
1	+	3	CDSi	2731	-	3039	19.88	2731	-	3039	309
1	+	4	CDSi	3260	-	3417	11.01	3260	-	3415	156
1	+	5	CDSl	3500	-	3635	9.57	3501	-	3635	135
1	+		PolA	3748			-4.40				

Figure S4. Original and revised sequences for FaWRKY51A.2

Figure S5. Gene structure of strawberry WRKY family

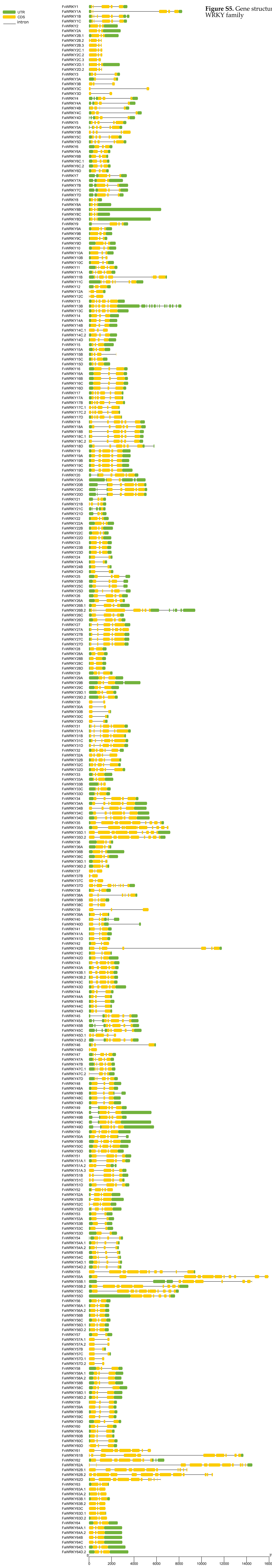

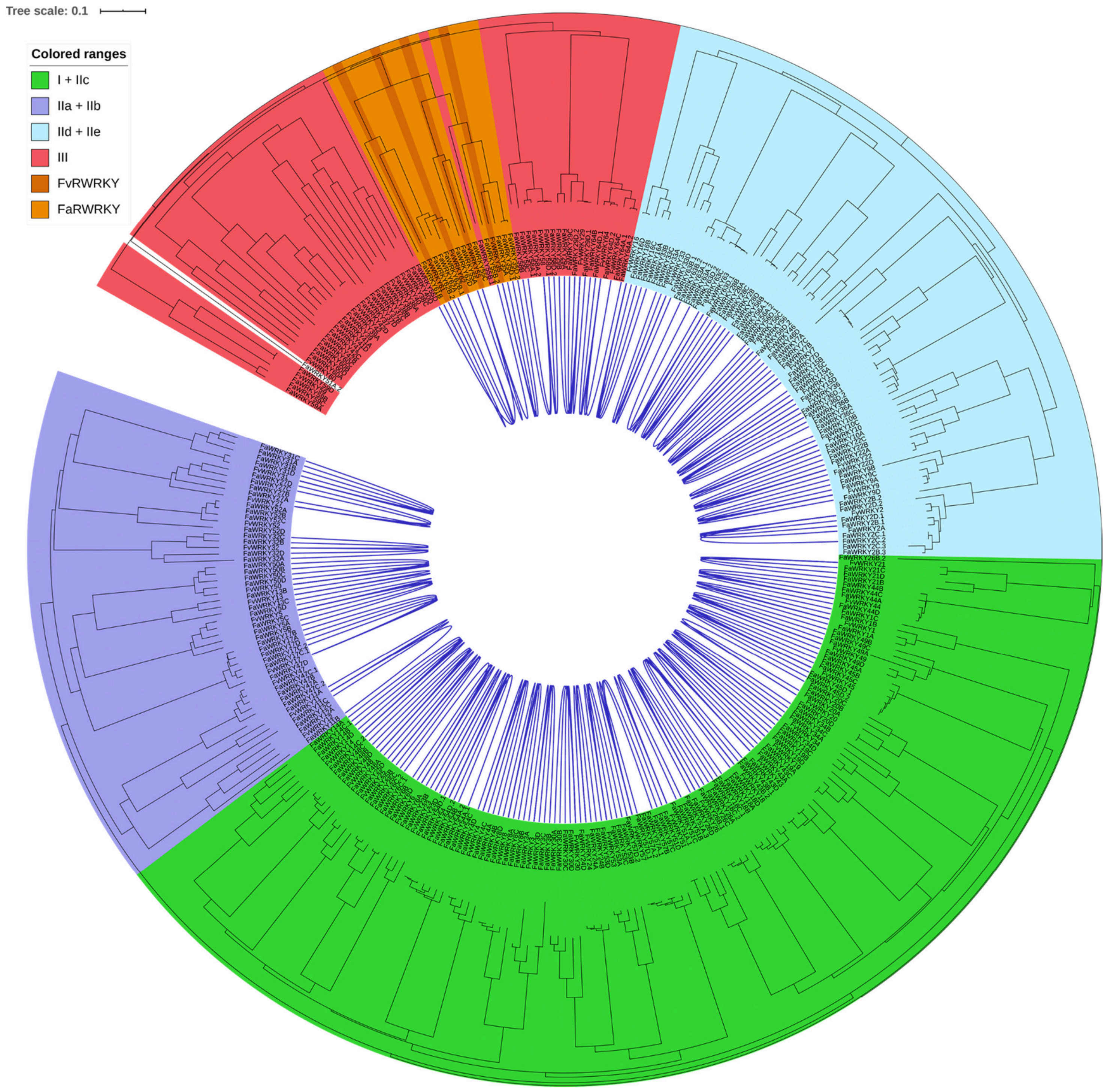

Figure S6. Phylogenetic analysis of *Fragaria vesca* (Fv) and *Fragaria x ananassa* (Fa) WRKY proteins. WRKY proteins are clustered into Groups I+IIc, IIa+IIb, IId+IIe and III. R protein-WRKY are clustered within their respective groups. The tree was inferred using the Neighbor-Joining (1000 bootstrap replicates) and drawn to scale, with branch lengths in the same units as those of the evolutionary distances used to infer the phylogenetic tree. The evolutionary distances were computed using the p-distance method and are in the units of the number of amino acid differences per site. All positions with less than 95% site coverage were eliminated. Connecting lines (blue) represent the shared synteny between Fv and Fa WRKY genes.

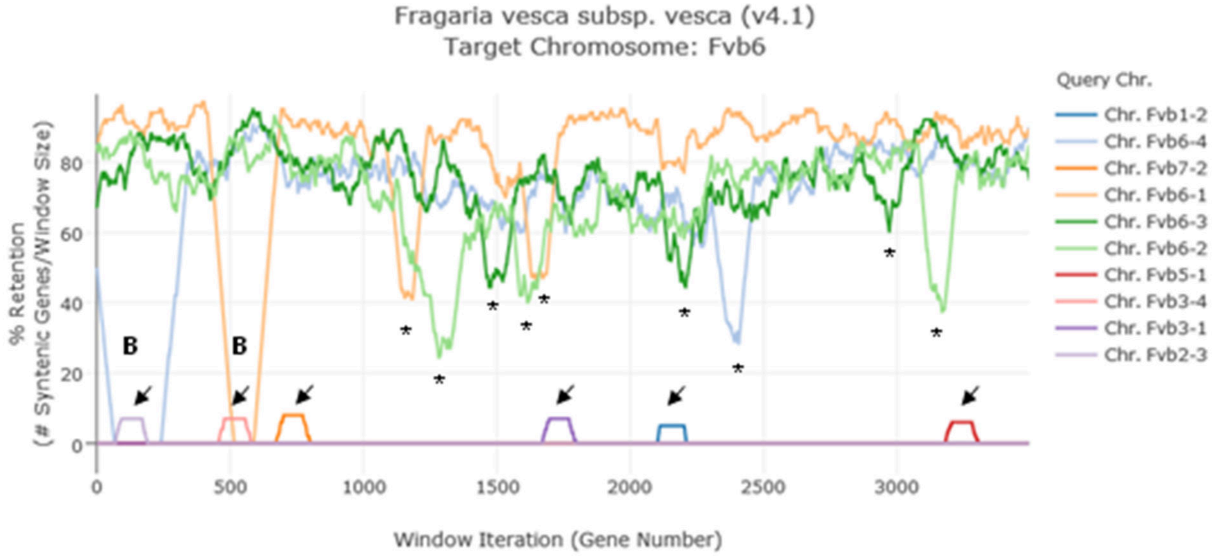

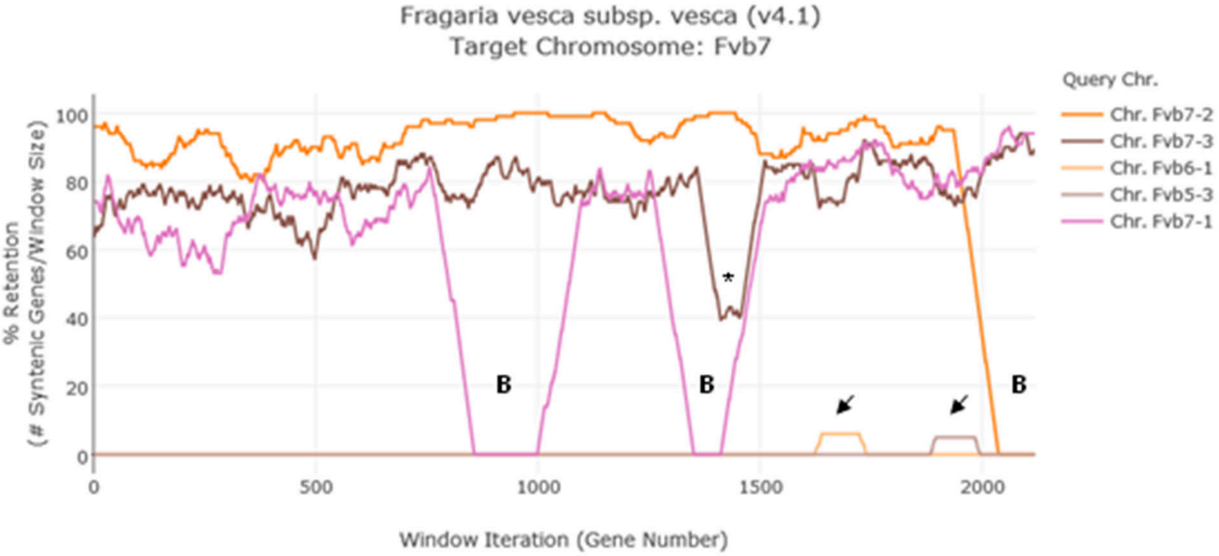

Figure S7. Gene retention and fractionation bias in Chromosomes 6 and 7 from *Fragaria vesca* and *Fragaria x ananassa* cv. Camarosa syntenic genes (1:4 syntenic depth). Whereas similar fractionation patterns are widespread in Fa chromosomes, group III FaWRKY loci, principally located in 6 and 7, seems to have been particularly affected by these events. Different regions within chromosomes have different levels of gene retention (fractionation), biased in some subgenomes (marked by "B"). Asterisks indicate areas of overfractionation. Syntenic regions with non-homologous chromosomes, potentially resulting from ectopic recombination, are indicated by arrows.

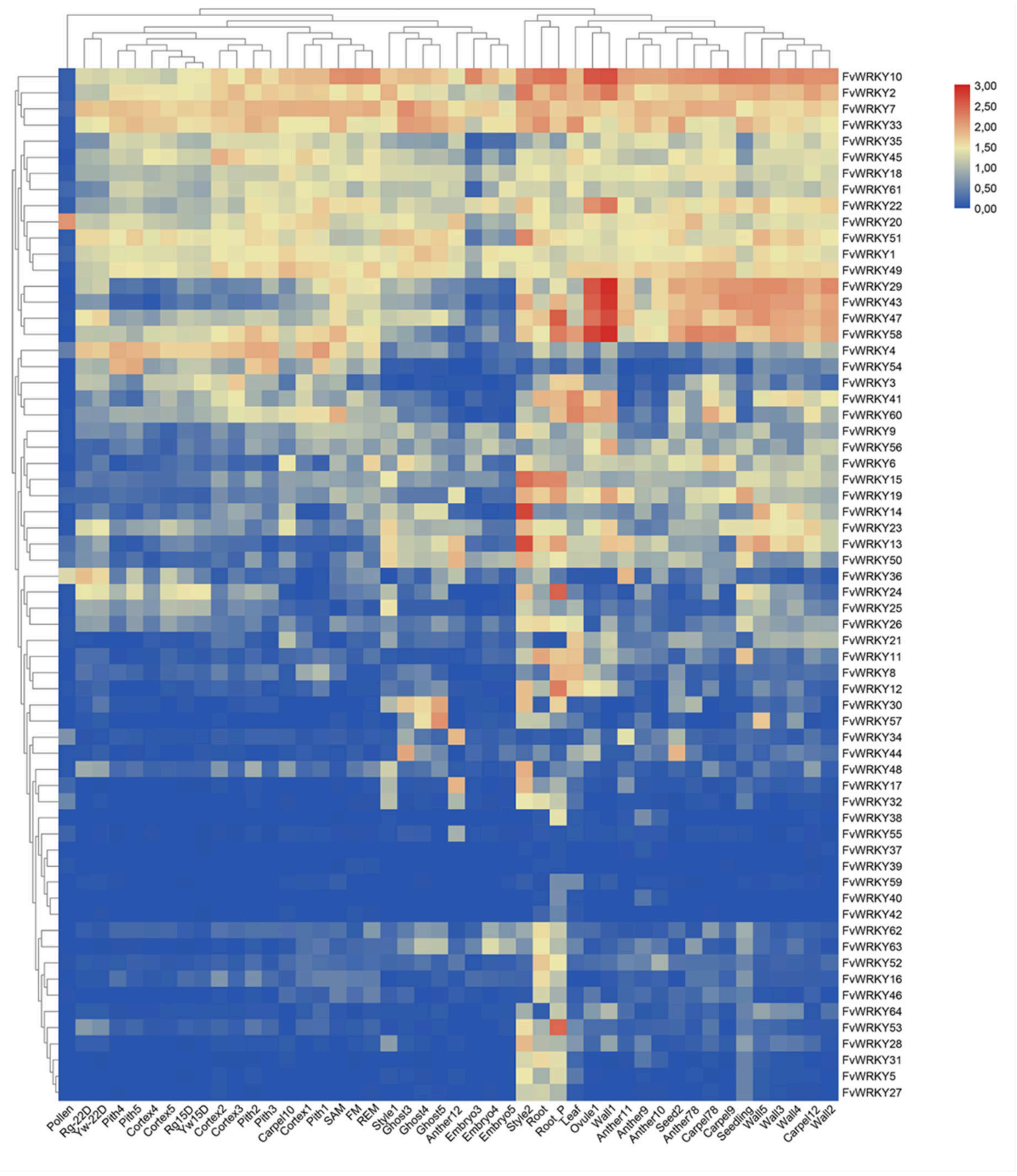

3,00
2,50
2,00
1,50
1,00
0,50
0,00
FvWRKY10
FvWRKY2
FvWRKY7
FvWRKY33
FvWRKY35
FvWRKY45
FvWRKY18
FvWRKY61
FvWRKY22
FvWRKY20
FvWRKY51
FvWRKY1
FvWRKY49
FvWRKY29
FvWRKY43
FvWRKY47
FvWRKY58
FvWRKY4
FvWRKY54
FvWRKY3
FvWRKY41
FvWRKY60
FvWRKY9
FvWRKY56
FvWRKY6
FvWRKY15
FvWRKY19
FvWRKY14
FvWRKY23
FvWRKY13
FvWRKY50
FvWRKY36
FvWRKY24
FvWRKY25
FvWRKY26
FvWRKY21
FvWRKY11
FvWRKY8
FvWRKY12
FvWRKY30
FvWRKY57
FvWRKY34
FvWRKY44
FvWRKY48
FvWRKY17
FvWRKY32
FvWRKY38
FvWRKY55
FvWRKY37
FvWRKY39
FvWRKY59
FvWRKY40
FvWRKY42
FvWRKY62
FvWRKY63
FvWRKY52
FvWRKY16
FvWRKY46
FvWRKY64
FvWRKY53
FvWRKY28
FvWRKY31
FvWRKY5
FvWRKY27
Pollen
Rg-22D
Yw-22D
Pith4
Pith5
Cortex4
Cortex5
Rg15D
Yw15D
Cortex2
Cortex3
Pith2
Pith3
Carpel10
Cortex1
Pith1
SAM
FM
REM
Style1
Ghost3
Ghost4
Ghost5
Anther12
Embryo3
Embryo4
Embryo5
Style2
Root
Root_P
Leaf
Ovule1
Wall1
Anther11
Anther9
Anther10
Seed2
Anther78
Carpel78
Carpel9
Seedling
Wall5
Wall3
Wall4
Carpel12
Wall2

Figure S8. Expression profiles of *FvWRKY* family members in different tissues, developmental stages and growth conditions of *F. vesca*. Color scale represents the expression level as log transformed TPM (Transcripts per Million) values. Abbreviations: SAM (shoot apical meristem), FM (floral meristem), REM (receptacle meristem), Anther7-8 (identified by stomium development and appearance of a preliminary lobed structure), Anther9 (microspore mother cells start meiosis), Anther10 (microspores are loose in the locule after callose wall holding tetrads disaggregates), Anther11 (pollen mitotic division occurs), anther12 (no visible change in anther development), Carpel7/8 (round carpel primordial reach the receptacle apex), Carpel9 (bowling pin shaped carpel primordial), Carpel10 (carpel is dived in almost equal apical and basal part by a central constriction), Carpel11 (style is elongated and became twice in length than the ovary base), Carpel12 (carpels have music note shape and styles are separated from each other), Cortex1 and Pith1 (flower just opened), Cortex2 and Pith2 (at about 3 DPA, when pollination occurs), Cortex3 and Pith3 (at about 6 DPA), Cortex4 and Pith4 (at about 9 DPA), Cortex5 and Pith5 (at about 12 DPA), Rg15D and Rg22D (Ruegen F7–4 receptacle tissue at 15 DPA and at 22 DPA, corresponding to green and white-turning stages respectively), Yw15D and Yw22D (Yellow Wonder 5AF7 receptacle tissue at 15 DPA and at 22 DPA, corresponding to green and white-turning stages respectively), Embryo3 and Ghost3 (embryo and seed without embryo inside at about 6 DPA characterized by heart shape), Embryo4 and Ghost4 (at about 9DPA, with immature cotyledons), Embryo5 and Ghost5 (ad about 12 DPA, mature embryo which fill up entire ovules), Leaf (young trifoliate leaves), Ovule1 and Pollen (collected from just open flower), Seed2 (complete achene from mature fruit), Seedling (complete seedling at 10 days post germination), Style1 (style and stigma from just open flowers), Style2 (style from flower at about 3 DPA), Wall1 (carpel wall from just open flower), Wall2 (carpel wall at about 3 DPA), Wall3 (carpel wall at about 6 DPA), Wall4 (carpel wall at about 9 DPA), Wall5 (carpel wall at about 12 DPA), Root (collected from 7 week old plants grown in aerated hydroponic culture) and Root_P (after 2 days of inoculation with *Phytophthora cactorum*). DPA: days post-anthesis.

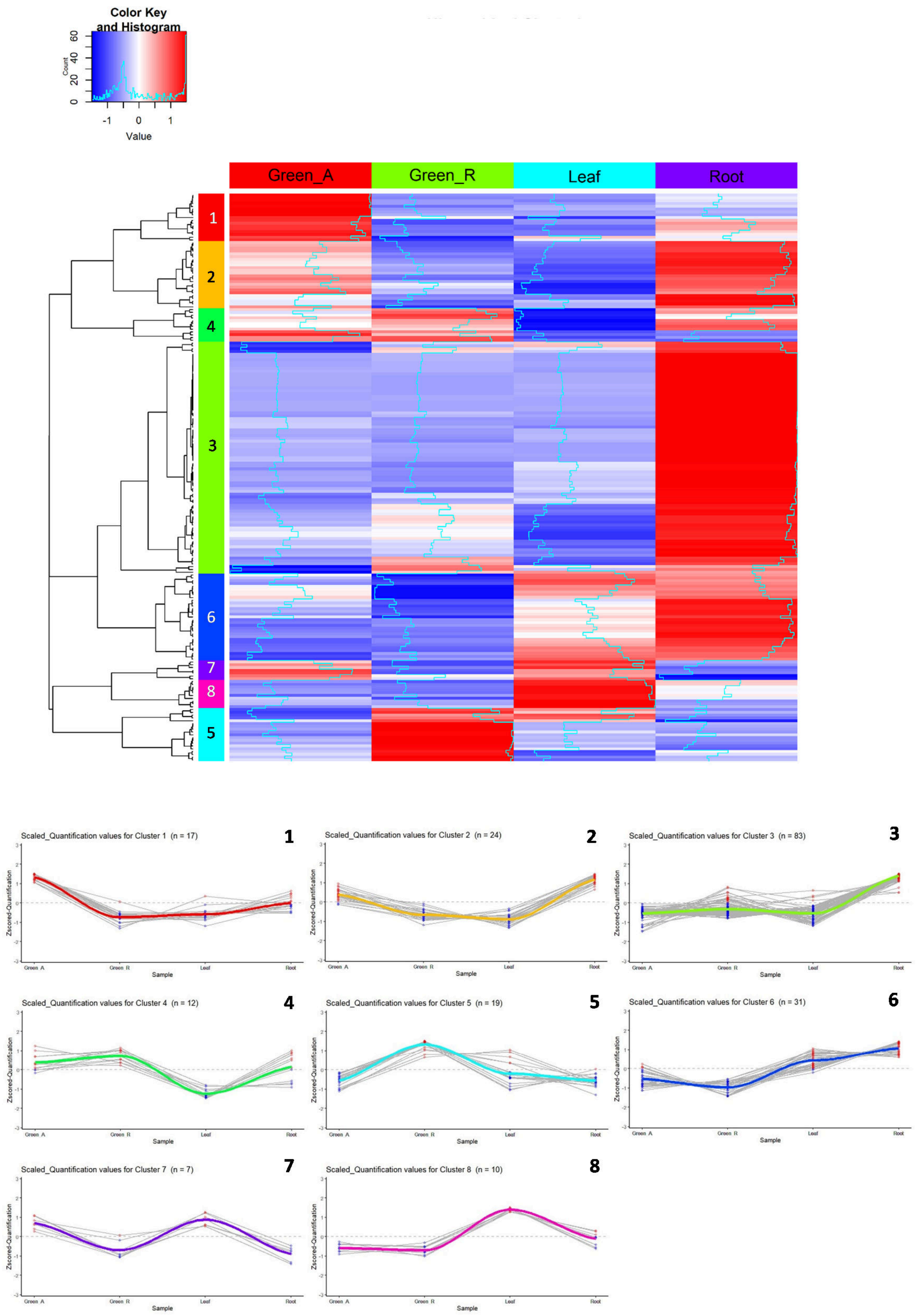

Figure S9. Expression and hierarchical clustering of *FaWRKY* genes in strawberry achene and receptacle of green fruits (Green_A and Green_R, respectively), leaves and root tissues. Heatmap represents gene expression ranged in a color scale from lowest (blue) to highest (red). Expression profiles of the statistically different groups (clusters) were depicted using same colors and numbers as in heatmap.

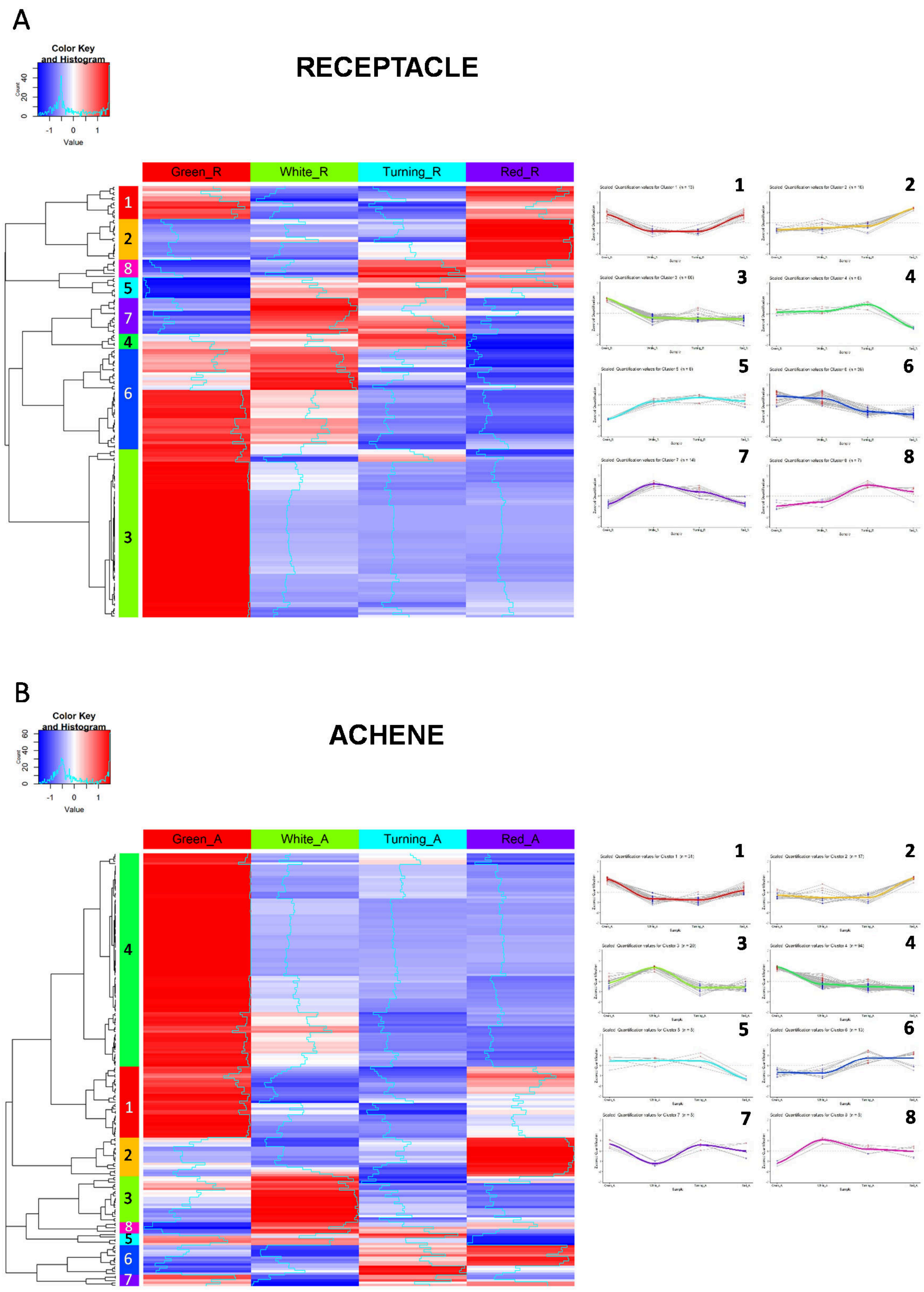

Figure S10. Expression and hierarchical clustering of *FaWRKY* genes during strawberry fruit ripening in receptacle (**A**) and achene (**B**) tissues. Heatmap represents gene expression ranged in a color scale from lowest (blue) to highest (red). Expression profiles of the statistically different (clusters) were depicted using same colors and numbers as in heatmap. Fruit ripening tissues are named Green_R, White_R, Turning_R, and Red_R (receptacles); Green_A, White_A, Turning_A, and Red_A (achenes).

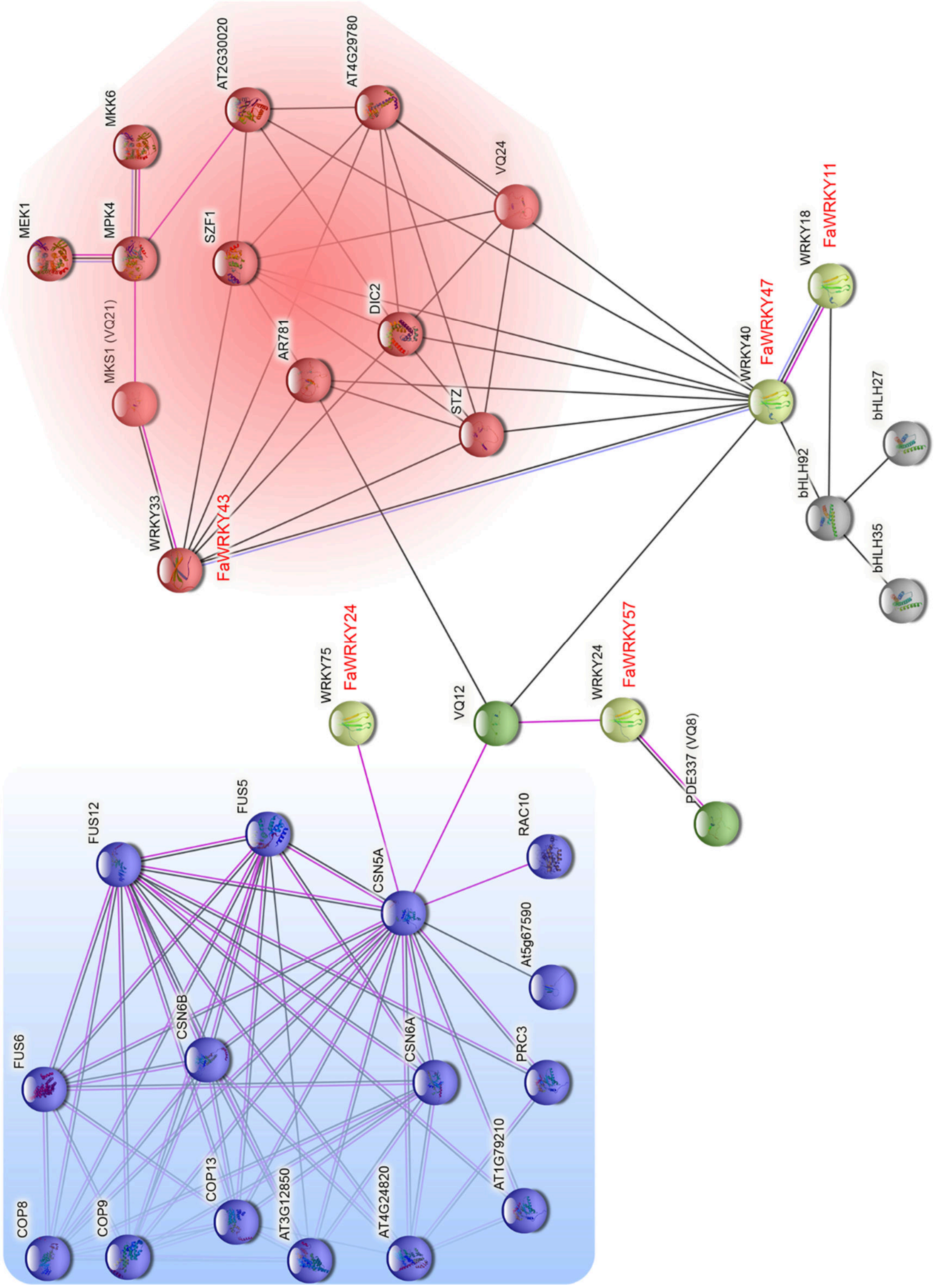

Figure S11. Interaction network constructed with the differentially expressed FaWRKYs during ripening (shown in red font), based on experimental and co-expression data with high confidence (0.700) from their *Arabidopsis* orthologs (black font). A cluster of proteins related to the proteasome, and developmental processes (blue area and balls) and a cluster of proteins involved in the biotic and abiotic stress responses (red area and balls) were identified to interact with FaWRKY24, FaWRKY47, and FaWRKY57. Also, FaWRKY11 and FaWRKY 47 interact with bHLH members (grey balls). The disconnected nodes (non-interacting FaWRKYs) were filtered and are not shown. Magenta, black and blue color lines indicate interactions experimentally-determined, co-expression and protein homology, respectively.

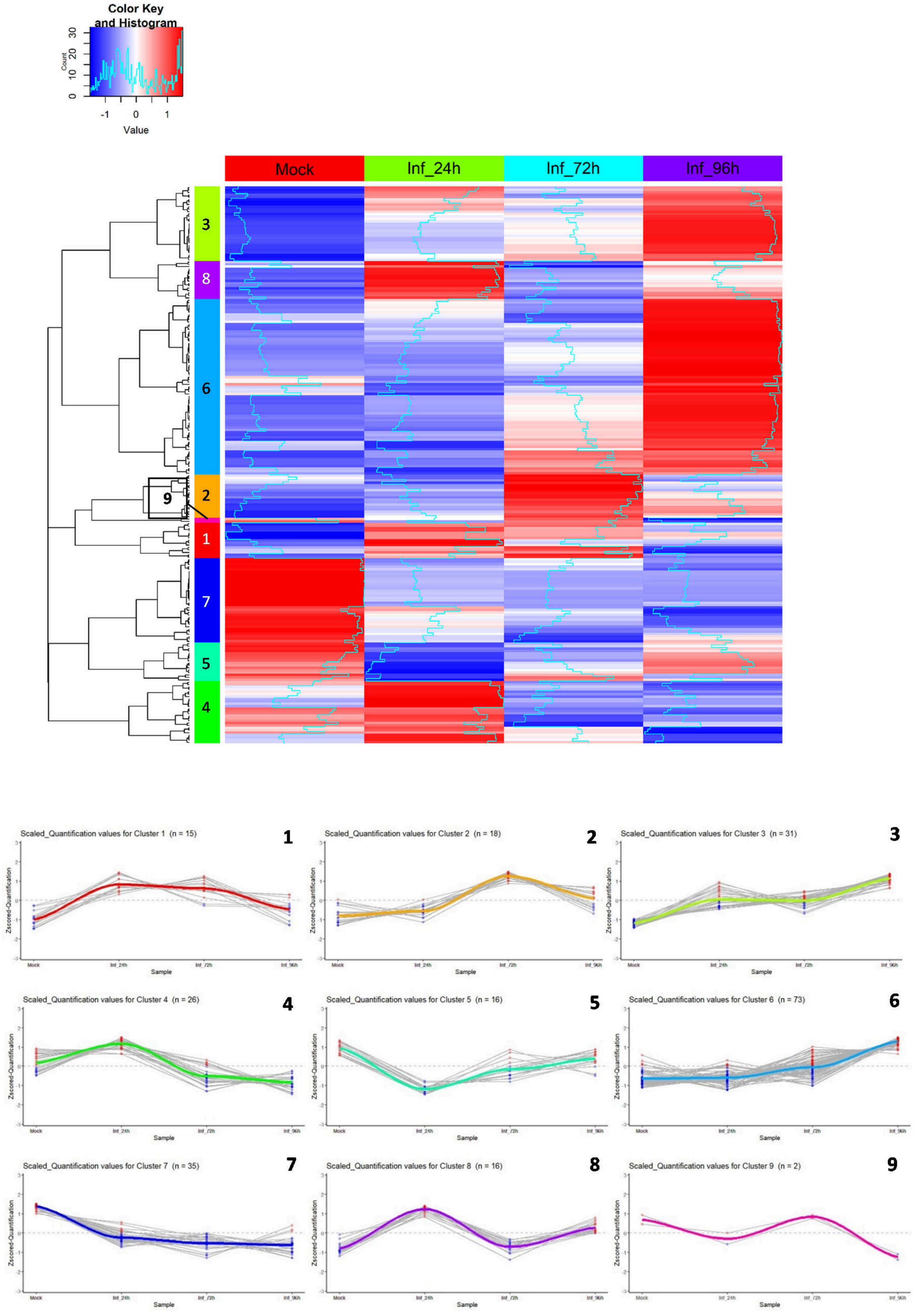

Figure S12. Expression and hierarchical clustering of *FaWRKY* genes in strawberry leaves, inoculated with mock or *C. fructicola* spores and collected at 24, 72 and 96 hours (Mock, Inf_24h, Inf_72h, and Inf_96h, respectively). Heatmap represents gene expression ranged in a color scale from lowest (blue) to highest (red). Expression profiles of the statistically different groups (clusters) were depicted using same colors and numbers as in heatmap.
