# Supplementary material for: A Comprehensive Study of the WRKY Transcription Factor Family in Strawberry
Source: Plants (Basel). 2022 Jun 15;11(12):1585. doi: 10.3390/plants11121585 (PMC9229891; doi:10.3390/plants11121585)
Supplement: Supplementary file 1 [file plants-11-01585-s001.zip › Table S19 candidate genes.pdf]

Table S19. A selection of putative candidate *FaWRKY* genes for further studies. The gene name is only shown when the number of times that the specific motif appears within the promoter region of the gene is greater than an arbitrary threshold (ThN) displayed in the corresponding header line, so it is considered a "true" sensitive gene. This does not mean that genes with lower values are not sensitive. + and – signals, represent the presence in lower number of times and absence, respectively, of the specific *cis*-acting motifs, and are displayed to aid evaluation of each gene. More information can be found in Tables S10. ABRE, Aux, MeJA, SA, ET, are abscisic acid, auxin, methyl jasmonate, salicylic acid, and ethylene-responsive elements, respectively. Biotic stress responsive elements are WUN-motif, WRE3, box S, W box, MYB-like sequence. Abiotic stress responsive elements are MBS, CCAAT-box, STRE, LTR, ARE, GC-motif. Red and dark blue colours, are outstanding up- and down-regulated ripening genes from data in Figure 9. Orange and light blue colours, are outstanding up- and down-regulated defense genes from data in Figure 10. One asterisk, up- or down-regulated genes both in ripening and defense processes from data in Figures 9 and 10. Two asterisks, genes containing more than 3 predicted gibberellin-responsive cis-elements (see Table S10). †, *WRKY* genes from the STRING analysis. It is worth to mention that although *WRKY30A/B/D* genes showed an outstanding up-regulation in defense (see Figure 10), their promoter regions are not enriched enough in *cis*-acting elements to be included within this table.

**HORMONE**					**STRESS**	
**ABRE** (ThN= 5)	**Aux** (ThN= 3)	**MeJA** (ThN= 7)	**SA** (ThN= 2)	**ET** (ThN= 3)	**Biotic** (ThN= 6)	**Abiotic** (ThN= 8)
1A	-	+	-	-	1A	+
+	-	+	2A	+	2A	+
-	-	+	2B.1	+	+	2B.1
+	+	2B.2	-	-	2B.2	2B.2
+	2B.3	2B.3	-	-	2B.3	2B.3
-	2C.3	+	2C.3	-	2C.3	2C.3
2D.1	+	+	-	-	2D.1	+
2D.2	+	+	-	-	+	2D.2
+	-	+	-	+	+	3B
+	+	3D	-	+	+	+
-	-	-	-	-	4B	+
-	-	+	-	4C	+	+
+	-	+	+	-	+	5B
5C	-	5C	-	-	5C	5C
5D	+	+	+	+	+	5D
6A	-	+	+	+	+	6A
6B	-	+	6B	+	+	+
6C.1	-	+	+	+	+	6C.1
6C.2	-	+	+	+	+	6C.2
6D	-	+	+	+	+	+
7A	-	+	-	+	+	7A
7C	-	+	-	-	+	7C
7D	-	+	-	7D	+	7D
+	-	+	-	-	+	8A

8B	+	+	-	-	8B	8B
+	+	+	-	-	+	8C
8D	-	+	-	-	+	8D
+	9B	+	-	+	+	+
+	+	+	+	+	+	9D
10A	+	10A	-	+	+	+
+	-	10B	-	+	+	+
+	+	+	-	+	+	10C
+	-	11B†	-	11B†	+	+
12A	12A	12A	-	+	+	+
12C	12C	12C	+	+	+	+
13B	13B	13B	-	+	+	+
13C	-	+	-	-	13C	+
14A	-	+	-	+	+	+
14C.1	-	+	-	+	+	+
14C.2	-	+	-	+	+	+
14D	-	+	-	-	+	+
+	-	+	+	+	15A	+
+	-	-	15B	+	15B	15B
+	-	+	-	15C	15C	15B
+	-	+	-	+	15D	+
-	+	-	-	+	+	16D
17A*	+	+	-	-	+	+
17B	+	+	-	-	+	+
17C.1	+	+	-	-	+	+
17C.2	+	+	-	-	+	+
17D	+	+	-	-	+	+
+	-	-	+	+	+	18A
-	-	+	+	+	+	18B
+	-	+	+	+	+	18C.1
+	-	+	+	+	18C.2	18C.2
+	-	+	+	+	18D	18D
+	-	+	-	19A	+	+
+	+	-	-	-	19C	+
+	-	+	-	+	20A	+
+	-	+	+	+	20B	+
-	-	-	-	+	+	20C
21B	+	+	+	-	21B	21B
+	+	-	21C	+	+	21C
-	+	-	+	+	+	21D
-	+	-	-	+	+	22A
22B	+	22B	+	-	22B	+
+	+	+	+	-	22C	+
+	-	+	-	-	23B	+
23D	-	+	+	-	23D	+

24A†	-	+	-	+	+	+
24B†	-	+	-	-	+	+
24D†	-	+	-	-	+	+
25B	+	+	-	-	+	+
25D	-	+	-	-	+	+
+	-	26A	26A	+	26A	26A
26D	-	-	-	+	26D	26D
27B	-	+	+	-	+	+
27C	-	+	-	-	+	+
28A	28A	+	28A	-	+	+
28B	28B	+	+	-	+	+
+	-	+	28C	+	+	+
+	-	+	28D	+	+	28D
-	+	+	-	+	+	29A
29B	+	+	-	+	+	+
-	-	+	-	+	+	29D.2*
+	+	+	31A	+	31A	31A
31B	31B	+	+	+	+	+
32A	+	+	-	-	+	32A
+	-	-	32C	+	+	+
32D	+	-	-	-	+	+
+	-	+	-	33A	+	+
+	-	+	-	-	+	34A
+	+	+	-	-	+	34B
34C	-	+	+	-	34C	34C
+	-	+	+	-	+	34D
+	-	-	35A	-	+	35A
+	-	-	-	35D.2	+	+
+	+	-	36A	+	36A	+
36B	-	-	-	-	+	+
+	+	+	+	+	36C	+
36D.1	-	+	+	-	+	36D.1
36D.2	-	+	+	-	+	+
+	-	+	37B	-	+	37B
37D	37D	-	-	-	+	+
+	+	38A	+	-	38A	38A
+	-	+	38B	-	+	+
39A	-	39A	+	-	+	39A
+	-	+	+	-	+	40D
41A	-	+	-	-	+	41A
41D	+	+	-	-	41D	41D
+	-	+	-	-	+	42B
42C	-	42C	-	-	+	+
42D	-	42D	-	-	+	+
43A†	-	+	-	-	+	+

43B.1†	+	43B.1†	+	-	43B.1†	+
43B.2†	+	+	+	-	43B.2†	+
43C†	+	+	-	-	+	+
43D†	+	+	+	+	+	+
+	44C	+	+	-	+	44C
45C	-	+	-	45C	+	+
45D.1	+	+	45D.1	-	+	45D.1
-	+	+	+	+	46D	+
+	+	47A†	-	+	+	+
47B†	-	+	+	-	+	47B†
+	+	+	-	-	+	47C.2†
47D†	+	+	+	+	47D†	+
48A	-	+	+	-	48A	+
48B	+	+	-	-	+	48B
48C	-	+	+	-	+	+
48D	-	+	-	-	48D	+
+	+	49A	-	+	49A	+
49B	+	+	-	+	+	49B
+	-	50B	-	-	+	+
+	+	+	-	-	+	50D
+	-	-	-	+	+	51A.1
51B	-	+	+	+	+	+
51D	-	+	+	-	+	+
+	-	+	-	+	+	52A
52C	-	+	-	+	+	52C
52D	-	+	-	+	+	+
+	+	53A	-	+	+	53A
53B*	-	+	-	-	53B*	+
+	-	+	-	-	53C	+
53D	-	+	-	+	+	+
54A.1	-	+	54A.1	+	54A.1	+
54A.2	-	+	54A.2	+	+	+
54B	-	+	54B	+	54B	+
+	-	-	+	+	54C	+
+	-	+	54D.1	+	+	+
+	-	+	54D.2	54D.2	+	+
+	+	55A	-	-	+	+
55B.1	-	55B.1	-	-	55B.1	55B.1
+	-	55B.2	+	+	+	55B.2
+	-	+	55C	-	+	55C
+	-	+	-	+	+	56B
+	-	+	56C	-	+	56C
+	-	+	-	-	+	56D.1
+	-	+	-	+	+	56D.2
57A.1†	-	-	57A.1†	-	+	57A.1†

57A.2†	-	+	+	+	+	57A.2†
57B†	-	57B†	+	-	+	57B†
57C†	-	-	-	-	+	+
57D.1**†	-	+	+	-	+	+
57D.2**†	-	+	+	-	+	+
+	-	+	+	+	+	58A.1
+	-	+	+	+	58A.2	58A.2
+	-	58C	58C	+	+	+
-	+	+	-	-	58D.1	+
+	+	+	-	+	58D.2	+
+	-	+	+	-	+	59A
+	-	+	+	-	+	59B
59C	-	+	+	-	+	+
-	-	-	-	+	59D	+
60A	-	60A	+	-	+	+
+	-	+	+	-	+	60B
60D	-	+	-	+	+	+
+	-	+	-	-	+	61B
62A	+	+	-	+	+	+
62B.1	-	62B.1	+	+	+	62B.1
+	63A.1	+	+	-	+	63A.1
+	+	+	+	-	+	63A.2
+	+	+	-	-	+	63B.1
+	+	+	-	-	+	63B.2
+	63C	+	+	-	63C	63C
63D.1	63D.1	+	+	-	+	+
+	+	+	+	-	+	63D.2
64C	-	+	-	-	+	64C
64D.1	-	+	+	-	64D.1	+
64D.2	-	+	+	-	+	+
